# Supplementary figures and images for: Integrated bioinformatics analysis of the NEDD4 family reveals a prognostic value of NEDD4L in clear-cell renal cell cancer
Source: PeerJ. 2021 Aug 17;9:e11880. doi: 10.7717/peerj.11880 (PMC8378337; doi:10.7717/peerj.11880)

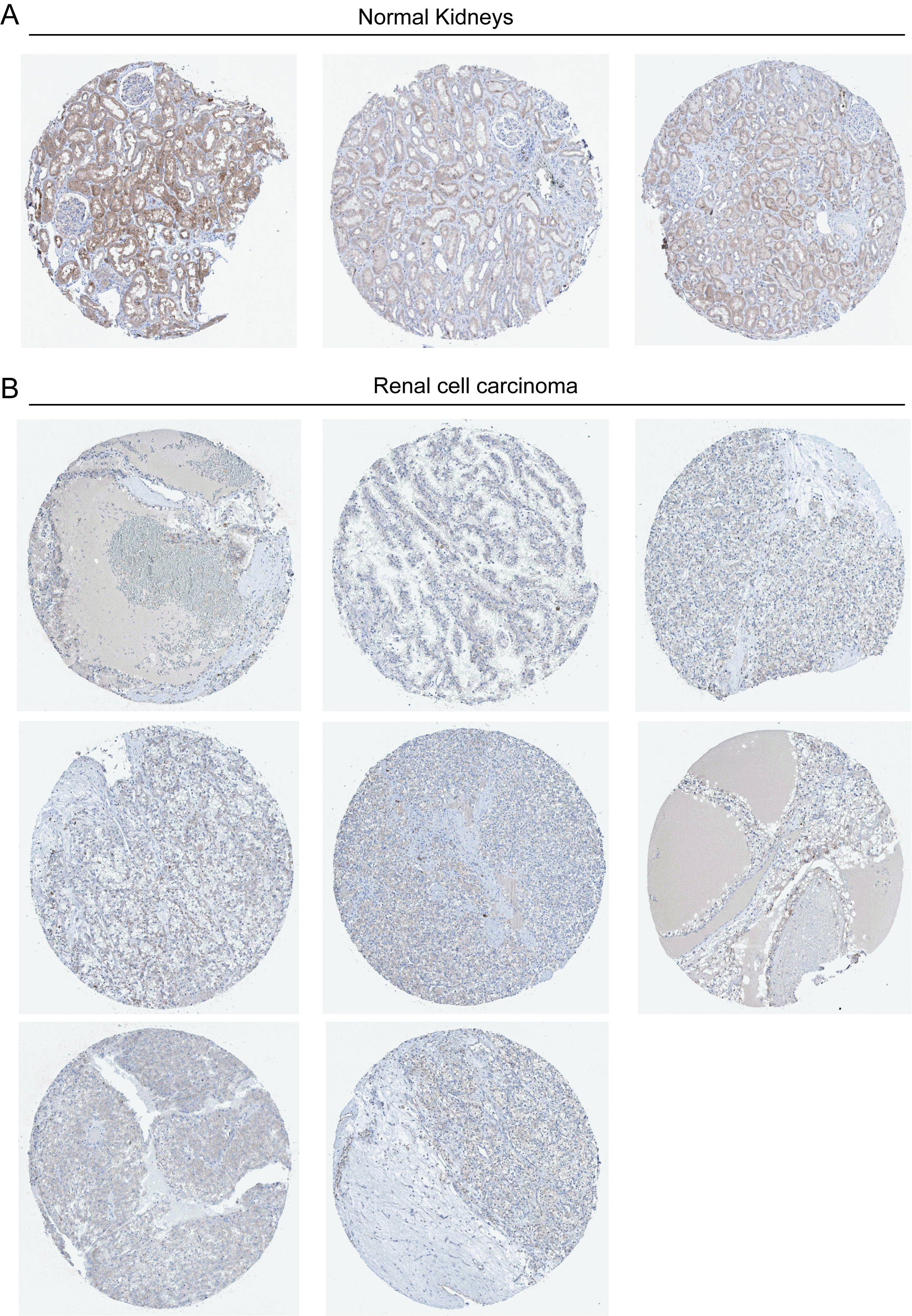

Supplement: Supplemental Information 1 [file peerj-09-11880-s001.jpg]

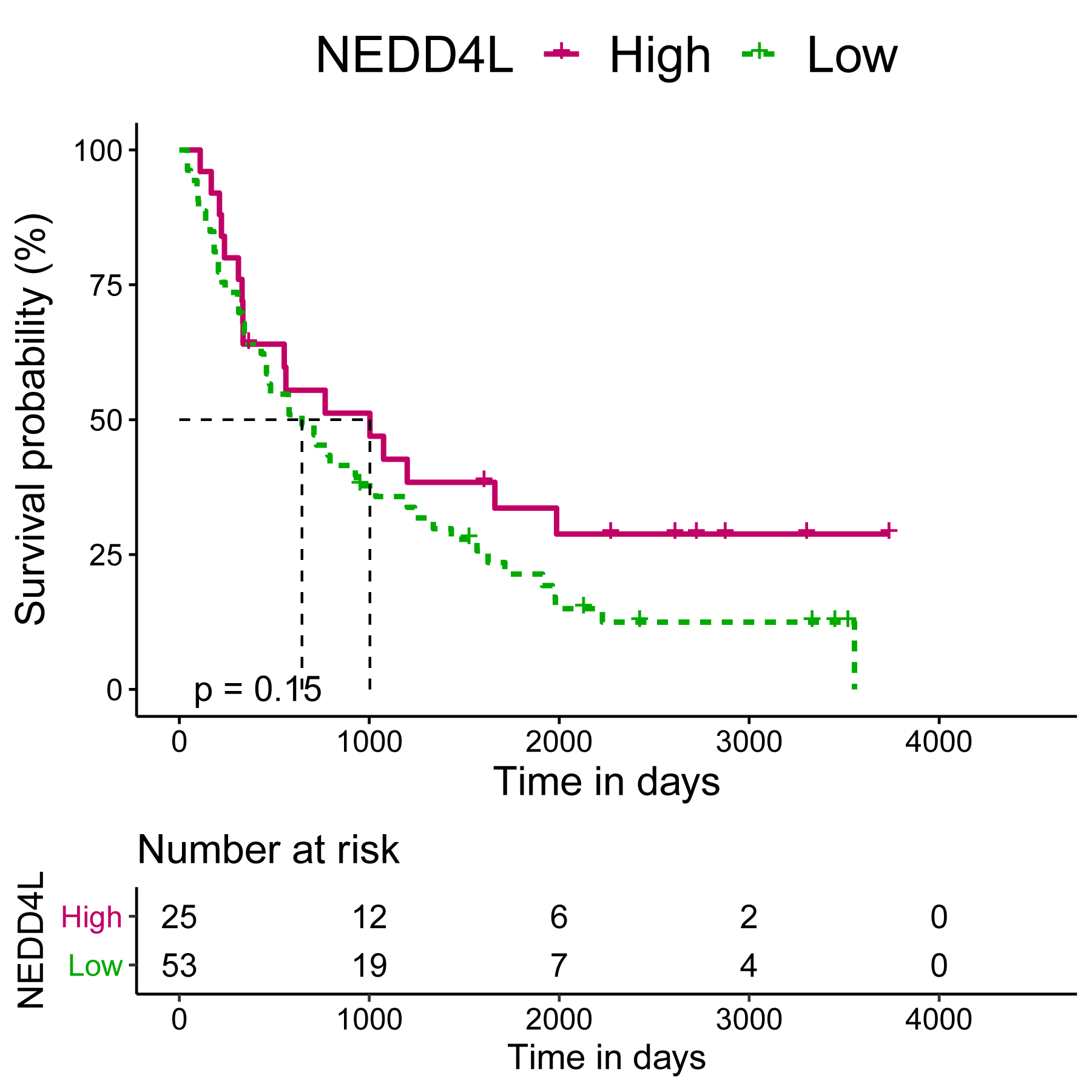

Supplement: Supplemental Information 2 [file peerj-09-11880-s002.png]

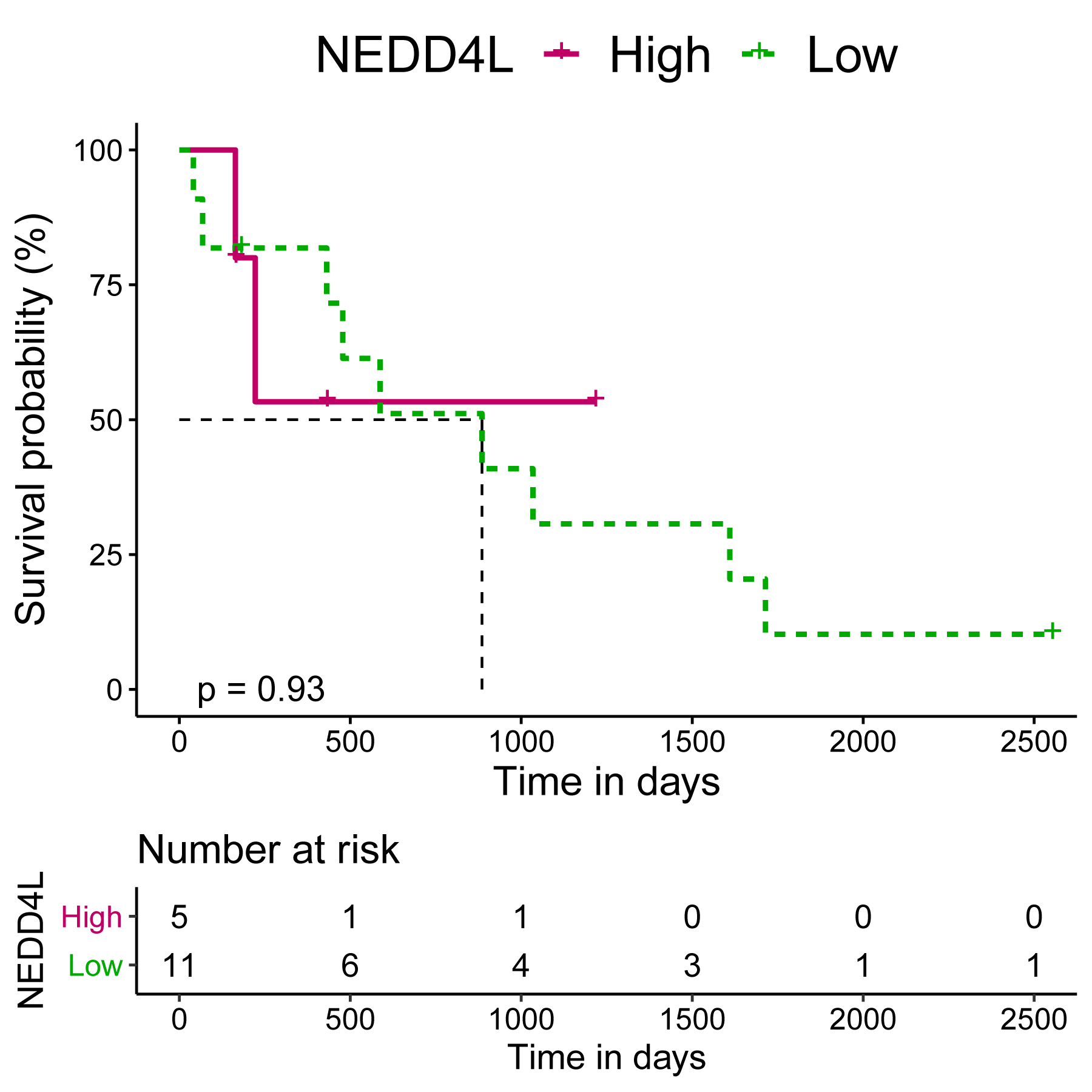

Supplement: Supplemental Information 3 [file peerj-09-11880-s003.png]

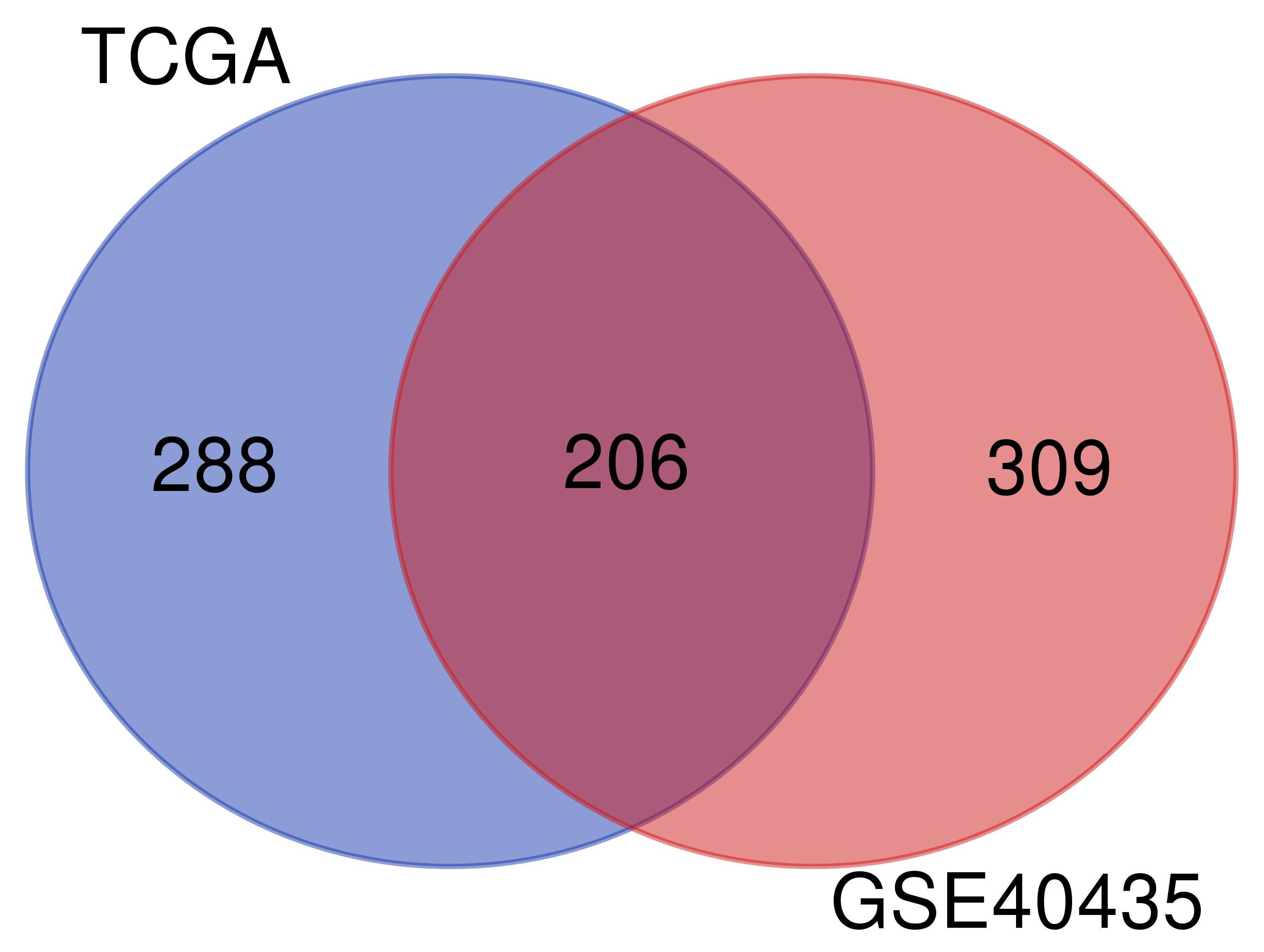

Supplement: Supplemental Information 4 [file peerj-09-11880-s004.png]

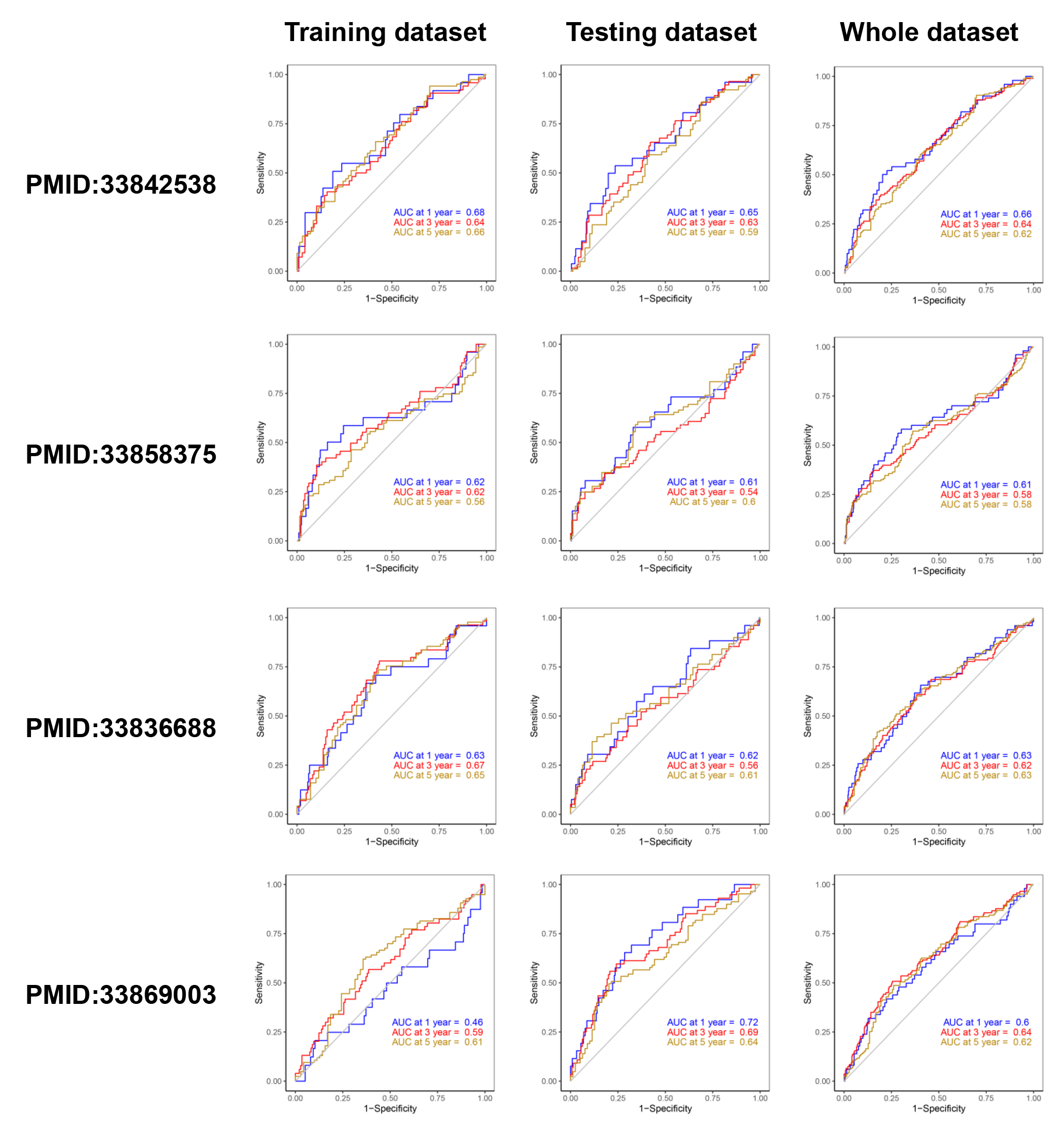

Supplement: Supplemental Information 5 [file peerj-09-11880-s005.png]
