# Supplementary material for: Integrated bioinformatics analysis of the NEDD4 family reveals a prognostic value of NEDD4L in clear-cell renal cell cancer
Source: PeerJ. 2021 Aug 17;9:e11880. doi: 10.7717/peerj.11880 (PMC8378337; doi:10.7717/peerj.11880)
Supplement: Supplemental Information 6 [file peerj-09-11880-s006.docx]

Supplementary table 1 Clinical information of patients

|  | sample | NEDD4L | OS.  time | Age | laterality | Histologic_grade | M_stage | N_stage | T_stage | Cancer_status | Gender | Tumor_stage |
| --- | --- | --- | --- | --- | --- | --- | --- | --- | --- | --- | --- | --- |
| 1 | TCGA.3Z.A93Z.01A | low | 385 | 69 | Right | G2 | M0 | N0 | T1a | TUMOR FREE | male | stage i |
| 2 | TCGA.6D.AA2E.01A | high | 362 | 68 | Right | G2 | MX | NX | T1b | TUMOR FREE | female | stage i |
| 3 | TCGA.A3.3306.01A | high | 1120 | 67 | Left | G3 | M0 | N0 | T1b |  | male | stage i |
| 4 | TCGA.A3.3307.01A | high | 1436 | 66 | Right | G3 | M0 | N0 | T3b |  | male | stage iii |
| 5 | TCGA.A3.3308.01A | high | 16 | 77 | Right | G2 | M0 | N0 | T3b | TUMOR FREE | female | stage iii |
| 6 | TCGA.A3.3311.01A | high | 1191 | 57 | Right | G2 | M0 | NX | T1 | TUMOR FREE | male | stage i |
| 7 | TCGA.A3.3313.01A | low | 735 | 59 | Right | G3 | M0 | N0 | T1b | TUMOR FREE | male | stage i |
| 8 | TCGA.A3.3316.01A | low | 1493 | 57 | Left | G3 | M0 | NX | T2 | TUMOR FREE | male | stage ii |
| 9 | TCGA.A3.3317.01A | high | 1491 | 67 | Left | G2 | M0 | N0 | T2 | WITH TUMOR | male | stage ii |
| 10 | TCGA.A3.3319.01A | low | 1130 | 70 | Left | G2 | M0 | NX | T1b | TUMOR FREE | male | stage i |
| 11 | TCGA.A3.3320.01A | high | 1508 | 52 | Right | G1 | M0 | NX | T1b | TUMOR FREE | female | stage i |
| 12 | TCGA.A3.3322.01A | high | 1478 | 51 | Left | G2 | M0 | NX | T1a | TUMOR FREE | male | stage i |
| 13 | TCGA.A3.3323.01A | low | 1106 | 53 | Right | G1 | M0 | NX | T1b | TUMOR FREE | male | stage i |
| 14 | TCGA.A3.3324.01A | high | 1186 | 51 | Right | G3 | M0 | NX | T1b | TUMOR FREE | male | stage i |
| 15 | TCGA.A3.3325.01A | high | 1170 | 52 | Right | G2 | M0 | NX | T1a | TUMOR FREE | male | stage i |
| 16 | TCGA.A3.3326.01A | high | 1137 | 47 | Right | G1 | M0 | NX | T1a | TUMOR FREE | male | stage i |
| 17 | TCGA.A3.3328.01A | high | 1385 | 79 | Left | G2 | M0 | N0 | T1b | TUMOR FREE | male | stage i |
| 18 | TCGA.A3.3329.01A | high | 1624 | 75 | Right | G2 | M0 | N0 | T1b | TUMOR FREE | male | stage i |
| 19 | TCGA.A3.3331.01A | high | 1257 | 86 | Right | G2 | M0 | N0 | T1 | TUMOR FREE | female | stage i |
| 20 | TCGA.A3.3335.01A | high | 1886 | 41 | Right | G4 | M0 | N0 | T2a | TUMOR FREE | male | stage ii |
| 21 | TCGA.A3.3343.01A | high | 945 | 79 | Left | G3 | M0 | N0 | T2 | TUMOR FREE | male | stage ii |
| 22 | TCGA.A3.3346.01A | low | 137 | 68 | Left | G3 | M0 | NX | T1b | WITH TUMOR | male | stage i |
| 23 | TCGA.A3.3347.01A | low | 1610 | 76 | Right | G2 | M0 | N1 | T1b | WITH TUMOR | female | stage iii |
| 24 | TCGA.A3.3349.01A | high | 1385 | 34 | Left | G2 | M0 | N0 | T1b | TUMOR FREE | female | stage i |
| 25 | TCGA.A3.3351.01A | high | 910 | 42 | Right | G2 | M0 | N0 | T2a | TUMOR FREE | male | stage ii |
| 26 | TCGA.A3.3352.01A | high | 561 | 74 | Left | G3 | M0 | N0 | T3a | TUMOR FREE | male | stage iii |
| 27 | TCGA.A3.3357.01A | low | 2688 | 62 | Right | G3 | M0 | N0 | T2 | TUMOR FREE | male | stage ii |
| 28 | TCGA.A3.3358.01A | low | 1307 | 57 | Left | G2 | M0 | N0 | T1a | TUMOR FREE | female | stage i |
| 29 | TCGA.A3.3359.01A | high | 2504 | 82 | Left | G2 | M0 | N0 | T1a | TUMOR FREE | female | stage i |
| 30 | TCGA.A3.3362.01A | high | 1559 | 60 | Left | G2 | M0 | N0 | T1a | TUMOR FREE | female | stage i |
| 31 | TCGA.A3.3363.01A | low | 319 | 50 | Left | G2 | M0 | N0 | T2 | TUMOR FREE | male | stage ii |
| 32 | TCGA.A3.3365.01A | high | 873 | 46 | Right | G2 | M0 | NX | T1a |  | male | stage i |
| 33 | TCGA.A3.3367.01A | high | 2270 | 72 | Left | G3 | M0 | N0 | T1b | TUMOR FREE | male | stage i |
| 34 | TCGA.A3.3370.01A | high | 2274 | 48 | Left | G2 | M0 | N0 | T1b | TUMOR FREE | female | stage i |
| 35 | TCGA.A3.3372.01A | high | 735 | 64 | Left | G2 | M0 | NX | T3 | TUMOR FREE | male | stage iii |
| 36 | TCGA.A3.3373.01A | high | 1621 | 54 | Right | G3 | M0 | N0 | T1b | TUMOR FREE | female | stage i |
| 37 | TCGA.A3.3374.01A | high | 1314 | 51 | Right | G2 | M0 | N0 | T1b |  | female | stage i |
| 38 | TCGA.A3.3376.01A | low | 1696 | 51 | Left | G2 | M0 | N0 | T1a | TUMOR FREE | male | stage i |
| 39 | TCGA.A3.3378.01A | high | 630 | 60 | Left | G3 | M0 | N0 | T1 | TUMOR FREE | male | stage i |
| 40 | TCGA.A3.3380.01A | high | 567 | 54 | Left | G2 | M0 | N0 | T1 | TUMOR FREE | male | stage i |
| 41 | TCGA.A3.3382.01A | low | 574 | 69 | Left | G3 | M0 | NX | T1b | WITH TUMOR | male | stage i |
| 42 | TCGA.A3.3383.01A | low | 861 | 52 | Left | G2 | M0 | NX | T1 |  | male | stage i |
| 43 | TCGA.A3.3385.01A | high | 1993 | 46 | Left | G2 | M0 | N0 | T1a | TUMOR FREE | female | stage i |
| 44 | TCGA.A3.3387.01A | low | 617 | 49 | Left | G2 | M0 | N0 | T1a | TUMOR FREE | male | stage i |
| 45 | TCGA.A3.A6NI.01A | high | 1018 | 47 | Left | G3 | MX | NX | T1a | TUMOR FREE | male | stage i |
| 46 | TCGA.A3.A6NJ.01A | low | 468 | 57 | Left | G1 | MX | NX | T1a | TUMOR FREE | female | stage i |
| 47 | TCGA.A3.A6NL.01A | high | 689 | 49 | Left | G2 | MX | NX | T1b | TUMOR FREE | female | stage i |
| 48 | TCGA.A3.A6NN.01A | high | 3 | 78 | Left | G2 | MX | NX | T1a |  | male | stage i |
| 49 | TCGA.A3.A8CQ.01A | high | 3 | 59 | Left | G2 | MX | NX | T1a |  | female | stage i |
| 50 | TCGA.A3.A8OV.01A | high | 340 | 75 | Right | G2 | MX | NX | T1a | TUMOR FREE | male | stage i |
| 51 | TCGA.A3.A8OW.01A | high | 323 | 37 | Left | G2 | MX | NX | T3a | TUMOR FREE | male | stage iii |
| 52 | TCGA.AK.3425.01A | low | 3343 | 68 | Left | G2 | M0 | N0 | T1 | TUMOR FREE | male | stage i |
| 53 | TCGA.AK.3426.01A | low | 885 | 37 | Right | G3 | M0 | N1 | T3a |  | male | stage iii |
| 54 | TCGA.AK.3427.01A | high | 3583 | 65 | Left | GX | M0 | N0 | T1a | TUMOR FREE | male | stage i |
| 55 | TCGA.AK.3428.01A | high | 3728 | 62 | Left | G2 | M0 | N0 | T3b | TUMOR FREE | male | stage iii |
| 56 | TCGA.AK.3429.01A | low | 3328 | 54 | Right | G2 | M0 | N0 | T2 | TUMOR FREE | female | stage ii |
| 57 | TCGA.AK.3431.01A | low | 2241 | 62 | Right | G3 | M0 | NX | T2 | TUMOR FREE | female | stage ii |
| 58 | TCGA.AK.3433.01A | high | 3409 | 48 | Right | GX | M0 | N0 | T2 | TUMOR FREE | female | stage ii |
| 59 | TCGA.AK.3434.01A | high | 2087 | 72 | Right | G2 | M0 | NX | T1b | WITH TUMOR | male | stage i |
| 60 | TCGA.AK.3436.01A | low | 3331 | 40 | Left | G2 | M1 | N0 | T2 | TUMOR FREE | male | stage iv |
| 61 | TCGA.AK.3440.01A | high | 2865 | 58 | Left | G3 | M0 | NX | T1a | TUMOR FREE | male | stage i |
| 62 | TCGA.AK.3443.01A | high | 1423 | 45 | Right | GX | M0 | N0 | T2 | TUMOR FREE | male | stage ii |
| 63 | TCGA.AK.3445.01A | low | 2392 | 69 | Left | G3 | M0 | NX | T3a | TUMOR FREE | male | stage iii |
| 64 | TCGA.AK.3447.01A | high | 1217 | 83 | Left | G2 | M0 | NX | T2 | TUMOR FREE | male | stage ii |
| 65 | TCGA.AK.3450.01A | high | 1508 | 85 | Right | G2 | M0 | N0 | T1a | TUMOR FREE | female | stage i |
| 66 | TCGA.AK.3451.01A | low | 2868 | 48 | Left | G3 | M0 | N0 | T2 | TUMOR FREE | male | stage ii |
| 67 | TCGA.AK.3453.01A | low | 2531 | 58 | Right | G2 | M0 | NX | T2 |  | female | stage ii |
| 68 | TCGA.AK.3454.01A | low | 874 | 84 | Right | G3 | M0 | NX | T1b | TUMOR FREE | male | stage i |
| 69 | TCGA.AK.3455.01A | high | 683 | 71 | Right | G3 | M0 | NX | T3b |  | female | stage iii |
| 70 | TCGA.AK.3456.01A | low | 1143 | 48 | Right | G3 | M0 | N0 | T2 |  | male | stage ii |
| 71 | TCGA.AK.3458.01A | high | 1168 | 48 | Right | G3 | M0 | NX | T1b | TUMOR FREE | male | stage i |
| 72 | TCGA.AK.3460.01A | low | 2508 | 58 | Right | G2 | M0 | NX | T1a | TUMOR FREE | male | stage i |
| 73 | TCGA.AK.3461.01A | high | 2217 | 72 | Left | G2 | M0 | NX | T1a | TUMOR FREE | male | stage i |
| 74 | TCGA.AK.3465.01A | high | 369 | 71 | Left | GX | M0 | NX | T1b | TUMOR FREE | female | stage i |
| 75 | TCGA.AS.3777.01A | high | 1238 | 63 | Left |  | M0 | NX | T1a | TUMOR FREE | male | stage i |
| 76 | TCGA.AS.3778.01A | high | 43 | 35 | Right | G1 | M0 | NX | T1a |  | male | stage i |
| 77 | TCGA.B0.4688.01A | low | 101 | 46 | Left | G4 | M1 | N0 | T4 | WITH TUMOR | male | stage iv |
| 78 | TCGA.B0.4690.01A | low | 43 | 65 | Right | G3 | M1 | N0 | T4 | WITH TUMOR | male | stage iv |
| 79 | TCGA.B0.4691.01A | low | 139 | 55 | Left | G3 | M1 | N0 | T2 | WITH TUMOR | male | stage iv |
| 80 | TCGA.B0.4693.01A | low | 77 | 72 | Left | G4 | M0 | N0 | T3a | TUMOR FREE | female | stage iii |
| 81 | TCGA.B0.4694.01A | low | 106 | 72 | Left | G4 | M0 | NX | T3b | TUMOR FREE | male | stage iii |
| 82 | TCGA.B0.4696.01A | low | 866 | 58 | Left | G3 | M0 | N0 | T3a | WITH TUMOR | male | stage iii |
| 83 | TCGA.B0.4697.01A | low | 578 | 46 | Right | G4 | M1 | NX | T3b | WITH TUMOR | female | stage iv |
| 84 | TCGA.B0.4698.01A | high | 42 | 75 | Left | G4 | M0 | NX | T4 | WITH TUMOR | male | stage iv |
| 85 | TCGA.B0.4699.01A | high | 110 | 74 | Right | G4 | M1 | N0 | T4 |  | male | stage iv |
| 86 | TCGA.B0.4700.01A | low | 1980 | 60 | Right | G4 | M1 | NX | T4 | WITH TUMOR | male | stage iv |
| 87 | TCGA.B0.4701.01A | high | 238 | 66 | Left | G3 | M1 | N0 | T3a | WITH TUMOR | female | stage iv |
| 88 | TCGA.B0.4703.01A | low | 182 | 51 | Left | G4 | M1 | N0 | T3a | WITH TUMOR | male | stage iv |
| 89 | TCGA.B0.4706.01A | low | 65 | 61 | Left | G4 | M0 | NX | T3a | TUMOR FREE | male | stage iii |
| 90 | TCGA.B0.4707.01A | low | 600 | 63 | Left | G4 | M0 | NX | T3a | WITH TUMOR | male | stage iii |
| 91 | TCGA.B0.4710.01A | high | 1755 | 75 | Right | G3 | M0 | N0 | T3a | TUMOR FREE | female | stage iii |
| 92 | TCGA.B0.4712.01A | low | 1337 | 76 | Left | G3 | M1 | NX | T3a | WITH TUMOR | male | stage iv |
| 93 | TCGA.B0.4713.01A | low | 202 | 76 | Right | G2 | M0 | NX | T3b | WITH TUMOR | female | stage iii |
| 94 | TCGA.B0.4714.01A | low | 99 | 81 | Right | G3 | M1 | NX | T3b | WITH TUMOR | male | stage iv |
| 95 | TCGA.B0.4718.01A | high | 1778 | 57 | Left | G2 | M0 | NX | T3a | WITH TUMOR | male | stage iii |
| 96 | TCGA.B0.4810.01A | low | 478 | 47 | Left | G3 | M0 | N1 | T3a | WITH TUMOR | male | stage iii |
| 97 | TCGA.B0.4811.01A | low | 1417 | 48 | Left | G3 | M0 | N0 | T3a | WITH TUMOR | male | stage iii |
| 98 | TCGA.B0.4813.01A | low | 18 | 68 | Right | G3 | M0 | NX | T3b | TUMOR FREE | male | stage iii |
| 99 | TCGA.B0.4814.01A | high | 168 | 58 | Right | G3 | M1 | N0 | T4 | WITH TUMOR | male | stage iv |
| 100 | TCGA.B0.4815.01A | low | 1588 | 65 | Left | G4 | M0 | NX | T3a | WITH TUMOR | male | stage iii |
| 101 | TCGA.B0.4816.01A | low | 1371 | 49 | Left | G3 | M0 | N0 | T2 | WITH TUMOR | male | stage ii |
| 102 | TCGA.B0.4817.01A | low | 1019 | 81 | Right | G3 | M0 | N0 | T3c | TUMOR FREE | male | stage iii |
| 103 | TCGA.B0.4818.01A | high | 510 | 68 | Right | G3 | M0 | NX | T2 | WITH TUMOR | female | stage ii |
| 104 | TCGA.B0.4819.01A | low | 183 | 60 | Right | G4 | M1 | NX | T3b | TUMOR FREE | female | stage iv |
| 105 | TCGA.B0.4821.01A | low | 1230 | 68 | Left | G3 | M0 | N0 | T3b | WITH TUMOR | female | stage iii |
| 106 | TCGA.B0.4822.01A | low | 1111 | 78 | Right | G4 | M0 | NX | T2 | WITH TUMOR | male | stage ii |
| 107 | TCGA.B0.4823.01A | low | 454 | 88 | Right | G2 | M0 | N0 | T1a | TUMOR FREE | male | stage i |
| 108 | TCGA.B0.4824.01A | low | 1657 | 49 | Left | G3 | M0 | N0 | T1a | TUMOR FREE | female | stage i |
| 109 | TCGA.B0.4827.01A | low | 885 | 77 | Left | G4 | M0 | N0 | T3b | WITH TUMOR | female | stage iii |
| 110 | TCGA.B0.4828.01A | low | 307 | 79 | Left | G3 | M1 | NX | T2 | WITH TUMOR | male | stage iv |
| 111 | TCGA.B0.4833.01A | low | 2386 | 82 | Left | G2 | M0 | N0 | T1b | WITH TUMOR | female | stage i |
| 112 | TCGA.B0.4834.01A | high | 2090 | 49 | Right | G3 | M0 | N0 | T1a | TUMOR FREE | male | stage i |
| 113 | TCGA.B0.4836.01A | low | 1238 | 61 | Right | G3 | M1 | NX | T3b | WITH TUMOR | male | stage iv |
| 114 | TCGA.B0.4837.01A | low | 1378 | 63 | Left | G3 | M0 | N0 | T1b | WITH TUMOR | male | stage i |
| 115 | TCGA.B0.4838.01A | high | 834 | 69 | Right | G3 | M0 | N0 | T1b | TUMOR FREE | female | stage i |
| 116 | TCGA.B0.4839.01A | low | 1639 | 80 | Left | G2 | M0 | N0 | T1b | TUMOR FREE | female | stage i |
| 117 | TCGA.B0.4841.01A | low | 204 | 63 | Left | G3 | M1 | NX | T2 | WITH TUMOR | male | stage iv |
| 118 | TCGA.B0.4842.01A | low | 1724 | 73 | Right | G4 | M0 | N0 | T3a | WITH TUMOR | female | stage iii |
| 119 | TCGA.B0.4843.01A | low | 320 | 57 | Right | G3 | M0 | N0 | T3a | WITH TUMOR | male | stage iii |
| 120 | TCGA.B0.4844.01A | low | 313 | 60 | Left | G3 | M1 | NX | T3a | WITH TUMOR | male | stage iv |
| 121 | TCGA.B0.4845.01A | high | 1986 | 70 | Right | G2 | M1 | NX | T3a | WITH TUMOR | male | stage iv |
| 122 | TCGA.B0.4846.01A | high | 1200 | 52 | Right | G2 | M1 | N0 | T3a | WITH TUMOR | male | stage iv |
| 123 | TCGA.B0.4847.01A | low | 793 | 60 | Left | G3 | M1 | NX | T3a |  | male | stage iv |
| 124 | TCGA.B0.4848.01A | low | 883 | 54 | Left | G3 | M0 | NX | T3b | WITH TUMOR | male | stage iii |
| 125 | TCGA.B0.4849.01A | low | 69 | 51 | Left | G3 | M0 | NX | T3a | WITH TUMOR | male | stage iii |
| 126 | TCGA.B0.4852.01A | high | 1121 | 78 | Left | G2 | M0 | N0 | T2 | WITH TUMOR | female | stage ii |
| 127 | TCGA.B0.4945.01A | low | 2145 | 75 | Right | G2 | M0 | N0 | T1a | TUMOR FREE | female | stage i |
| 128 | TCGA.B0.5075.01A | low | 637 | 77 | Left | G2 | M0 | N0 | T3a | TUMOR FREE | female | stage iii |
| 129 | TCGA.B0.5077.01A | high | 1317 | 77 | Left | G3 | M0 | N0 | T1a | TUMOR FREE | male | stage i |
| 130 | TCGA.B0.5080.01A | low | 342 | 63 | Right | G3 | M1 | N0 | T3a | WITH TUMOR | male | stage iv |
| 131 | TCGA.B0.5081.01A | low | 362 | 79 | Right | G2 | M0 | N0 | T3b | WITH TUMOR | female | stage iii |
| 132 | TCGA.B0.5083.01A | high | 1045 | 63 | Left | G3 | M0 | N0 | T1a |  | male | stage i |
| 133 | TCGA.B0.5084.01A | high | 222 | 33 | Right | G3 | M1 | N1 | T3a | WITH TUMOR | male | stage iv |
| 134 | TCGA.B0.5085.01A | low | 770 | 76 | Left | G3 | M0 | N0 | T3a | TUMOR FREE | female | stage iii |
| 135 | TCGA.B0.5088.01A | low | 563 | 53 | Right | G3 | M0 | N0 | T1b | TUMOR FREE | male | stage i |
| 136 | TCGA.B0.5092.01A | low | 459 | 53 | Left | G3 | M1 | N0 | T1a | WITH TUMOR | female | stage iv |
| 137 | TCGA.B0.5094.01A | low | 333 | 62 | Left | G2 | M1 | N0 | T3b |  | male | stage iv |
| 138 | TCGA.B0.5095.01A | low | 245 | 81 | Right | G3 | M0 | N0 | T3a | TUMOR FREE | male | stage iii |
| 139 | TCGA.B0.5096.01A | low | 68 | 72 | Left | GX | M0 | N1 | T3a | TUMOR FREE | female | stage iii |
| 140 | TCGA.B0.5097.01A | low | 665 | 59 | Right | G2 | M0 | N0 | T3b | WITH TUMOR | female | stage iii |
| 141 | TCGA.B0.5098.01A | high | 1584 | 53 | Right | G3 | M0 | NX | T1 |  | female | stage i |
| 142 | TCGA.B0.5099.01A | low | 485 | 88 | Right | G3 | M0 | NX | T3b |  | female | stage iii |
| 143 | TCGA.B0.5100.01A | high | 1913 | 72 | Right | G3 | M0 | NX | T3a | WITH TUMOR | male | stage iii |
| 144 | TCGA.B0.5102.01A | high | 2764 | 74 | Left | G3 | M0 | NX | T1 |  | female | stage i |
| 145 | TCGA.B0.5106.01A | low | 1598 | 64 | Left | G2 | M0 | N0 | T1a | TUMOR FREE | male | stage i |
| 146 | TCGA.B0.5107.01A | low | 927 | 65 | Right | G4 | M1 | N0 | T2 | WITH TUMOR | female | stage iv |
| 147 | TCGA.B0.5108.01A | low | 1782 | 54 | Left | G2 | M0 | N0 | T3a | TUMOR FREE | male | stage iii |
| 148 | TCGA.B0.5109.01A | low | 587 | 69 | Left | G4 | M0 | N1 | T3b | WITH TUMOR | male | stage iii |
| 149 | TCGA.B0.5110.01A | high | 2009 | 71 | Right | G2 | M0 | N0 | T1a | TUMOR FREE | female | stage i |
| 150 | TCGA.B0.5113.01A | high | 1175 | 69 | Right | G2 | M0 | N0 | T3a | TUMOR FREE | female | stage iii |
| 151 | TCGA.B0.5115.01A | high | 1604 | 43 | Left | G3 | M1 | N0 | T2 | WITH TUMOR | male | stage iv |
| 152 | TCGA.B0.5116.01A | low | 1274 | 52 | Right | G3 | M0 | N0 | T3b | TUMOR FREE | male | stage iii |
| 153 | TCGA.B0.5117.01A | high | 1608 | 40 | Right | G2 | M0 | NX | T1b | TUMOR FREE | male | stage i |
| 154 | TCGA.B0.5119.01A | high | 1552 | 61 | Right | G2 | M0 | N0 | T1b | TUMOR FREE | female | stage i |
| 155 | TCGA.B0.5120.01A | low | 1169 | 72 | Left | G2 | M0 | N0 | T1a | TUMOR FREE | female | stage i |
| 156 | TCGA.B0.5121.01A | low | 1485 | 56 | Right | G2 | M0 | N0 | T1b | TUMOR FREE | male | stage i |
| 157 | TCGA.B0.5399.01A | high | 1411 | 46 | Right | G2 | M0 | N0 | T1b | TUMOR FREE | male | stage i |
| 158 | TCGA.B0.5400.01A | low | 1733 | 59 | Right | G4 | M0 | N0 | T3b | TUMOR FREE | female | stage iii |
| 159 | TCGA.B0.5402.01A | high | 1290 | 64 | Left | G4 | M0 | NX | T4 | WITH TUMOR | male | stage iv |
| 160 | TCGA.B0.5690.01A | high | 3392 | 53 | Right | G1 | M0 | NX | T1b | TUMOR FREE | female | stage i |
| 161 | TCGA.B0.5691.01A | high | 3431 | 66 | Left | G3 | M0 | N0 | T1a | WITH TUMOR | female | stage i |
| 162 | TCGA.B0.5692.01A | low | 3944 | 66 | Right | G3 | M0 | N0 | T3b | TUMOR FREE | female | stage iii |
| 163 | TCGA.B0.5693.01A | high | 4074 | 47 | Right | G2 | M0 | NX | T1b | TUMOR FREE | female | stage i |
| 164 | TCGA.B0.5694.01A | low | 480 | 71 | Left | G3 | M0 | N0 | T3a | WITH TUMOR | male | stage iii |
| 165 | TCGA.B0.5695.01A | high | 2150 | 61 | Left | G2 | M0 | N0 | T1b | TUMOR FREE | female | stage i |
| 166 | TCGA.B0.5696.01A | high | 2609 | 69 | Right | G4 | M0 | N0 | T3a | WITH TUMOR | male | stage iii |
| 167 | TCGA.B0.5697.01A | low | 2630 | 50 | Right | G2 | M0 | N0 | T1a | TUMOR FREE | male | stage i |
| 168 | TCGA.B0.5698.01A | low | 3631 | 77 | Right | G3 | M0 | N0 | T1b | TUMOR FREE | male | stage i |
| 169 | TCGA.B0.5699.01A | high | 3841 | 53 | Left | G2 | M0 | N0 | T1 | TUMOR FREE | male | stage i |
| 170 | TCGA.B0.5700.01A | low | 1790 | 77 | Left | G2 | M0 | N0 | T1a | TUMOR FREE | male | stage i |
| 171 | TCGA.B0.5701.01A | high | 2461 | 65 | Left | G4 | M0 | N0 | T3b | WITH TUMOR | male | stage iii |
| 172 | TCGA.B0.5702.01A | high | 2172 | 71 | Left | G2 | M0 | N0 | T1b | TUMOR FREE | male | stage i |
| 173 | TCGA.B0.5703.01A | low | 2246 | 73 | Left | G3 | M0 | N0 | T1b | TUMOR FREE | male | stage i |
| 174 | TCGA.B0.5705.01A | high | 4537 | 65 | Left | G2 | M0 | N0 | T1 | TUMOR FREE | female | stage i |
| 175 | TCGA.B0.5706.01A | low | 3205 | 45 | Left | G2 | M0 | N0 | T2 | TUMOR FREE | male | stage ii |
| 176 | TCGA.B0.5707.01A | low | 3744 | 39 | Right | G3 | M0 | N0 | T1a | TUMOR FREE | female | stage i |
| 177 | TCGA.B0.5709.01A | high | 3974 | 62 | Right | G3 | M0 | NX | T3a | TUMOR FREE | female | stage iii |
| 178 | TCGA.B0.5710.01A | low | 2430 | 57 | Right | G2 | M0 | N0 | T1b | WITH TUMOR | male | stage i |
| 179 | TCGA.B0.5711.01A | high | 3989 | 50 | Right | G3 | M0 | NX | T3b | TUMOR FREE | male | stage iii |
| 180 | TCGA.B0.5712.01A | high | 2722 | 68 | Left | G3 | M1 | N0 | T2 | WITH TUMOR | female | stage iv |
| 181 | TCGA.B0.5713.01A | high | 2782 | 75 | Left | G3 | M0 | N0 | T3b | TUMOR FREE | female | stage iii |
| 182 | TCGA.B0.5812.01A | high | 3834 | 53 | Right | G3 | M0 | NX | T1b | TUMOR FREE | male | stage i |
| 183 | TCGA.B2.3923.01A | high | 992 | 59 | Right | G2 | M0 | NX | T2 | TUMOR FREE | male | stage ii |
| 184 | TCGA.B2.3924.01A | low | 1092 | 73 | Left | G2 | M0 | NX | T1b | TUMOR FREE | male | stage i |
| 185 | TCGA.B2.4098.01A | low | 51 | 72 | Left | G2 | M0 | NX | T1b | TUMOR FREE | female | stage i |
| 186 | TCGA.B2.4099.01A | high | 972 | 83 | Right | G3 | M0 | NX | T1a | TUMOR FREE | male | stage i |
| 187 | TCGA.B2.4101.01A | high | 648 | 52 | Left | G3 | M0 | NX | T2a | TUMOR FREE | male | stage ii |
| 188 | TCGA.B2.4102.01A | high | 952 | 61 | Right | G2 | M0 | NX | T1b | TUMOR FREE | male | stage i |
| 189 | TCGA.B2.5633.01A | high | 963 | 56 | Right | G2 | M0 | N0 | T1b | TUMOR FREE | male | stage i |
| 190 | TCGA.B2.5635.01A | low | 755 | 74 | Right | G2 | M0 | NX | T1a | TUMOR FREE | male | stage i |
| 191 | TCGA.B2.5636.01A | high | 919 | 79 | Right | G2 | M0 | NX | T1a | TUMOR FREE | male | stage i |
| 192 | TCGA.B2.5639.01A | high | 1003 | 46 | Left | G3 | M1 | NX | T3 | WITH TUMOR | male | stage iv |
| 193 | TCGA.B2.5641.01A | low | 656 | 79 | Left | G3 | M0 | N0 | T1a | TUMOR FREE | male | stage i |
| 194 | TCGA.B2.A4SR.01A | high | 507 | 61 | Right |  | M0 | NX | T2a | TUMOR FREE | male | stage ii |
| 195 | TCGA.B4.5377.01A | high | 365 | 68 | Right | G3 | M1 | N0 | T3 |  | female | stage iv |
| 196 | TCGA.B4.5378.01A | high | 175 | 62 | Right | G2 | M0 | N0 | T1 |  | male | stage i |
| 197 | TCGA.B4.5832.01A | low | 155 | 65 | Left | G2 | M0 | N0 | T3b |  | male | stage iii |
| 198 | TCGA.B4.5834.01A | high | 38 | 59 | Right | G1 | M0 | N0 | T1 |  | male | stage i |
| 199 | TCGA.B4.5835.01A | low | 16 | 64 | Left | G2 | M0 | N0 | T1 |  | female | stage i |
| 200 | TCGA.B4.5836.01A | high | 141 | 61 | Right | G2 | M0 | N0 | T1b |  | female | stage i |
| 201 | TCGA.B4.5838.01A | high | 166 | 52 | Left | G2 | M0 | N1 | T3 |  | male | not reported |
| 202 | TCGA.B4.5843.01A | high | 11 | 45 | Right | G2 | M0 | N0 | T1 |  | male | stage i |
| 203 | TCGA.B4.5844.01A | high | 7 | 61 | Right | G1 | M0 | N0 | T2 |  | female | stage ii |
| 204 | TCGA.B8.4143.01A | low | 709 | 66 | Left | G3 | M1 | N0 | T3a | WITH TUMOR | female | stage iv |
| 205 | TCGA.B8.4148.01A | high | 1520 | 63 | Left | G3 | M0 | N0 | T1a | TUMOR FREE | female | stage i |
| 206 | TCGA.B8.4151.01A | high | 1299 | 51 | Right | G2 | M0 | N0 | T3a | TUMOR FREE | female | stage iii |
| 207 | TCGA.B8.4154.01A | high | 1380 | 73 | Left | G2 | M0 | N0 | T1a | TUMOR FREE | female | stage i |
| 208 | TCGA.B8.4619.01A | high | 523 | 58 | Left | G2 | M0 | N0 | T1a | TUMOR FREE | male | stage i |
| 209 | TCGA.B8.4620.01A | low | 777 | 70 | Left | G2 | M0 | N0 | T3a | TUMOR FREE | female | stage iii |
| 210 | TCGA.B8.4621.01A | low | 788 | 63 | Left | G3 | M0 | N0 | T1b | TUMOR FREE | male | stage i |
| 211 | TCGA.B8.4622.01A | low | 1525 | 57 | Left | G3 | M1 | N0 | T3a | TUMOR FREE | male | stage iv |
| 212 | TCGA.B8.5158.01A | high | 1218 | 56 | Left | G4 | M0 | N1 | T3a | TUMOR FREE | male | stage iii |
| 213 | TCGA.B8.5159.01A | high | 722 | 61 | Right | G3 | M0 | N0 | T1a | TUMOR FREE | female | stage i |
| 214 | TCGA.B8.5162.01A | low | 36 | 62 | Left | G2 | M0 | NX | T2a | TUMOR FREE | male | stage ii |
| 215 | TCGA.B8.5163.01A | low | 822 | 63 | Right | G3 | M0 | N0 | T3a |  | female | stage iii |
| 216 | TCGA.B8.5164.01A | high | 26 | 65 | Right | G3 | M0 | N0 | T3a | TUMOR FREE | male | stage iii |
| 217 | TCGA.B8.5165.01A | high | 737 | 43 | Right | G2 | M0 | N0 | T1a | TUMOR FREE | male | stage i |
| 218 | TCGA.B8.5545.01A | high | 1525 | 42 | Left | G2 | M0 | N0 | T1a | TUMOR FREE | male | stage i |
| 219 | TCGA.B8.5546.01A | high | 505 | 38 | Right | G2 | M0 | N0 | T1b | TUMOR FREE | female | stage i |
| 220 | TCGA.B8.5549.01A | low | 194 | 53 | Right | G3 | M0 | N0 | T1b | TUMOR FREE | male | stage i |
| 221 | TCGA.B8.5550.01A | high | 1476 | 71 | Right | G3 | M0 | N0 | T3a | TUMOR FREE | male | stage iii |
| 222 | TCGA.B8.5551.01A | low | 16 | 65 | Right | G3 | M0 | N0 | T1b | TUMOR FREE | female | stage i |
| 223 | TCGA.B8.5553.01A | high | 435 | 67 | Left | G2 | M0 | N0 | T1b | TUMOR FREE | female | stage i |
| 224 | TCGA.B8.A54D.01A | low | 830 | 69 | Left | G2 | MX | NX | T3a | TUMOR FREE | male | stage iii |
| 225 | TCGA.B8.A54E.01A | low | 909 | 62 | Right | G3 | MX | NX | T1b | TUMOR FREE | female | stage i |
| 226 | TCGA.B8.A54F.01A | low | 519 | 49 | Left | G2 | MX | NX | T1a | TUMOR FREE | female | stage i |
| 227 | TCGA.B8.A54G.01A | high | 53 | 50 | Right | G3 | MX | NX | T1a | TUMOR FREE | male | stage i |
| 228 | TCGA.B8.A54H.01A | high | 256 | 69 | Left | G3 | MX | N0 | T2a |  | female | stage ii |
| 229 | TCGA.B8.A54I.01A | low | 150 | 48 | Left | G3 | MX | NX | T1b | TUMOR FREE | male | stage i |
| 230 | TCGA.B8.A54J.01A | low | 528 | 60 | Right | G2 | MX | NX | T2a | TUMOR FREE | male | stage ii |
| 231 | TCGA.B8.A54K.01A | high | 469 | 61 | Right | G1 | MX | NX | T1a | TUMOR FREE | male | stage i |
| 232 | TCGA.B8.A7U6.01A | low | 495 | 54 | Left | G3 |  | NX | T1a | TUMOR FREE | female | stage i |
| 233 | TCGA.B8.A8YJ.01A | low | 431 | 60 | Right | G2 |  | NX | T1b | TUMOR FREE | female | stage i |
| 234 | TCGA.BP.4158.01A | low | 3377 | 69 | Right | G2 | M0 | N0 | T1b | TUMOR FREE | male | stage i |
| 235 | TCGA.BP.4159.01A | low | 2601 | 70 | Right | G2 | M0 | N0 | T1b | WITH TUMOR | male | stage i |
| 236 | TCGA.BP.4160.01A | low | 2881 | 67 | Left | G2 | M0 | N0 | T3a | TUMOR FREE | male | stage iii |
| 237 | TCGA.BP.4161.01A | high | 2746 | 74 | Right | G3 | M0 | NX | T1b | WITH TUMOR | male | stage i |
| 238 | TCGA.BP.4162.01A | high | 3074 | 65 | Right | G2 | M0 | N0 | T1b | TUMOR FREE | female | stage i |
| 239 | TCGA.BP.4163.01A | low | 2839 | 60 | Right | G3 | M0 | N0 | T3a | TUMOR FREE | female | stage iii |
| 240 | TCGA.BP.4164.01A | high | 992 | 51 | Left | G2 | M0 | NX | T3a | TUMOR FREE | female | stage iii |
| 241 | TCGA.BP.4165.01A | low | 3037 | 64 | Right | G1 | M0 | N0 | T1b | WITH TUMOR | female | stage i |
| 242 | TCGA.BP.4166.01A | low | 13 | 69 | Left | G3 | M0 | N0 | T3a | TUMOR FREE | male | stage iii |
| 243 | TCGA.BP.4167.01A | low | 2718 | 59 | Right | G2 | M0 | NX | T3a | TUMOR FREE | male | stage iii |
| 244 | TCGA.BP.4169.01A | high | 701 | 76 | Right | G2 | M0 | N0 | T2 | WITH TUMOR | female | stage ii |
| 245 | TCGA.BP.4170.01A | low | 2343 | 72 | Left | G2 | M0 | N0 | T1b | TUMOR FREE | female | stage i |
| 246 | TCGA.BP.4173.01A | low | 1893 | 47 | Right | G3 | M0 | N0 | T2 | TUMOR FREE | male | stage ii |
| 247 | TCGA.BP.4174.01A | high | 1879 | 49 | Right | G3 | M0 | N0 | T2 | TUMOR FREE | male | stage ii |
| 248 | TCGA.BP.4176.01A | low | 1955 | 64 | Right | G2 | M0 | NX | T1b | TUMOR FREE | male | stage i |
| 249 | TCGA.BP.4177.01A | high | 1670 | 65 | Right | G2 | M0 | NX | T1a | TUMOR FREE | male | stage i |
| 250 | TCGA.BP.4325.01A | high | 2964 | 64 | Left | G2 | M0 | N0 | T1b | TUMOR FREE | female | stage i |
| 251 | TCGA.BP.4326.01A | high | 1625 | 53 | Left | G2 | M0 | N0 | T1b | WITH TUMOR | female | stage i |
| 252 | TCGA.BP.4327.01A | high | 109 | 75 | Right | G2 | M0 | N0 | T2 | TUMOR FREE | female | stage ii |
| 253 | TCGA.BP.4329.01A | low | 845 | 75 | Right | G2 | M0 | N0 | T3a | WITH TUMOR | male | stage iii |
| 254 | TCGA.BP.4330.01A | high | 1888 | 60 | Left | G2 | M0 | N0 | T3a | TUMOR FREE | female | stage iii |
| 255 | TCGA.BP.4331.01A | high | 2454 | 52 | Left | G2 | M0 | N0 | T1a | TUMOR FREE | male | stage i |
| 256 | TCGA.BP.4332.01A | high | 1133 | 36 | Left | G2 | M0 | N0 | T3a | TUMOR FREE | male | stage iii |
| 257 | TCGA.BP.4334.01A | high | 645 | 56 | Right | G3 | M0 | N0 | T3a | WITH TUMOR | male | stage iii |
| 258 | TCGA.BP.4335.01A | low | 475 | 65 | Left | G3 | M1 | N0 | T3a | WITH TUMOR | female | stage iv |
| 259 | TCGA.BP.4337.01A | high | 2 | 76 | Left | G4 | M0 | N0 | T3b | TUMOR FREE | female | stage iii |
| 260 | TCGA.BP.4338.01A | low | 2859 | 43 | Left | G3 | M0 | N0 | T1b | WITH TUMOR | male | stage i |
| 261 | TCGA.BP.4340.01A | high | 562 | 70 | Right | G2 | M0 | N0 | T1b | TUMOR FREE | female | stage i |
| 262 | TCGA.BP.4341.01A | high | 1589 | 67 | Left | G2 | M0 | NX | T3a |  | male | stage iii |
| 263 | TCGA.BP.4342.01A | low | 2256 | 79 | Left | G3 | M0 | N0 | T2 | WITH TUMOR | male | stage ii |
| 264 | TCGA.BP.4343.01A | low | 1912 | 64 | Right | G3 | M0 | N0 | T3a | WITH TUMOR | male | stage iii |
| 265 | TCGA.BP.4344.01A | low | 1666 | 75 | Right | G2 | M0 | NX | T1a | TUMOR FREE | female | stage i |
| 266 | TCGA.BP.4345.01A | low | 1516 | 62 | Right | G3 | M0 | N0 | T3b | TUMOR FREE | male | stage iii |
| 267 | TCGA.BP.4346.01A | high | 1493 | 57 | Right | G3 | M0 | N0 | T3b | TUMOR FREE | male | stage iii |
| 268 | TCGA.BP.4347.01A | low | 1367 | 74 | Right | G2 | M0 | NX | T3b | TUMOR FREE | male | stage iii |
| 269 | TCGA.BP.4349.01A | low | 372 | 68 | Left | G2 | M0 | NX | T1a |  | female | stage i |
| 270 | TCGA.BP.4351.01A | high | 970 | 51 | Left | G2 | M0 | N0 | T3a | WITH TUMOR | female | stage iii |
| 271 | TCGA.BP.4352.01A | low | 344 | 74 | Right | G4 | M1 | N0 | T3b | WITH TUMOR | female | stage iv |
| 272 | TCGA.BP.4353.01A | low | 375 | 61 | Left | G2 | M0 | N0 | T1 | TUMOR FREE | male | stage i |
| 273 | TCGA.BP.4354.01A | low | 1034 | 40 | Left | G4 | M1 | N1 | T4 | WITH TUMOR | male | stage iv |
| 274 | TCGA.BP.4355.01A | low | 953 | 59 | Left | G4 | M0 | NX | T3a |  | female | stage iii |
| 275 | TCGA.BP.4756.01A | low | 374 | 62 | Right | G2 | M0 | N0 | T1b | TUMOR FREE | female | stage i |
| 276 | TCGA.BP.4758.01A | low | 2208 | 40 | Right | G2 | M0 | NX | T1a | TUMOR FREE | male | stage i |
| 277 | TCGA.BP.4759.01A | low | 2372 | 50 | Left | G2 | M0 | NX | T1a | TUMOR FREE | male | stage i |
| 278 | TCGA.BP.4760.01A | high | 2361 | 69 | Left | G2 | M0 | NX | T1a | WITH TUMOR | male | stage i |
| 279 | TCGA.BP.4761.01A | low | 182 | 57 | Right | G4 | M0 | N1 | T3a | TUMOR FREE | male | stage iii |
| 280 | TCGA.BP.4762.01A | low | 1343 | 42 | Left | G3 | M0 | NX | T1a | TUMOR FREE | male | stage i |
| 281 | TCGA.BP.4763.01A | low | 1270 | 79 | Right | G2 | M0 | NX | T1a | TUMOR FREE | female | stage i |
| 282 | TCGA.BP.4765.01A | high | 2184 | 43 | Right | G2 | M0 | NX | T1a | TUMOR FREE | male | stage i |
| 283 | TCGA.BP.4766.01A | high | 1462 | 43 | Right | G3 | M0 | NX | T1a | TUMOR FREE | female | stage i |
| 284 | TCGA.BP.4768.01A | high | 400 | 72 | Left | G2 | M0 | N0 | T1a | TUMOR FREE | female | stage i |
| 285 | TCGA.BP.4769.01A | high | 1876 | 63 | Left | G2 | M0 | NX | T1a | TUMOR FREE | male | stage i |
| 286 | TCGA.BP.4770.01A | low | 329 | 73 | Left | G4 | M0 | N0 | T4 | WITH TUMOR | female | stage iv |
| 287 | TCGA.BP.4771.01A | low | 162 | 62 | Right | G4 | M1 | N0 | T3a | WITH TUMOR | male | stage iv |
| 288 | TCGA.BP.4774.01A | high | 1885 | 57 | Right | G2 | M0 | NX | T1a | TUMOR FREE | female | stage i |
| 289 | TCGA.BP.4775.01A | high | 1843 | 55 | Left | G2 | M0 | NX | T1a |  | female | stage i |
| 290 | TCGA.BP.4776.01A | low | 411 | 52 | Left | G2 | M0 | NX | T1a | TUMOR FREE | male | stage i |
| 291 | TCGA.BP.4777.01A | low | 1731 | 46 | Right | G3 | M0 | NX | T1a | TUMOR FREE | male | stage i |
| 292 | TCGA.BP.4781.01A | low | 2080 | 78 | Right | G3 | M0 | NX | T1a | TUMOR FREE | male | stage i |
| 293 | TCGA.BP.4782.01A | high | 354 | 55 | Left | G2 | M0 | NX | T1a |  | female | stage i |
| 294 | TCGA.BP.4784.01A | high | 1854 | 67 | Right | G2 | M0 | NX | T1a | TUMOR FREE | female | stage i |
| 295 | TCGA.BP.4787.01A | low | 480 | 59 | Left | G4 | M1 | N0 | T3a | WITH TUMOR | female | stage iv |
| 296 | TCGA.BP.4789.01A | high | 1489 | 48 | Right | G2 | M0 | NX | T1a | TUMOR FREE | male | stage i |
| 297 | TCGA.BP.4790.01A | high | 1111 | 76 | Right | G2 | M0 | NX | T1a | TUMOR FREE | male | stage i |
| 298 | TCGA.BP.4795.01A | high | 620 | 74 | Right | G2 | M0 | N0 | T1a | TUMOR FREE | female | stage i |
| 299 | TCGA.BP.4797.01A | high | 1107 | 34 | Right | G3 | M0 | N0 | T3b | TUMOR FREE | male | stage iii |
| 300 | TCGA.BP.4798.01A | high | 334 | 74 | Left | G4 | M1 | N0 | T3b | WITH TUMOR | male | not reported |
| 301 | TCGA.BP.4799.01A | low | 1133 | 70 | Right | G3 | M0 | N0 | T3b | WITH TUMOR | male | stage iii |
| 302 | TCGA.BP.4801.01A | high | 1124 | 57 | Left | G2 | M0 | NX | T1a | TUMOR FREE | male | stage i |
| 303 | TCGA.BP.4803.01A | high | 204 | 79 | Right | G3 | M0 | NX | T3a | TUMOR FREE | male | stage iii |
| 304 | TCGA.BP.4804.01A | low | 1459 | 59 | Right | G2 | M0 | NX | T1b | WITH TUMOR | male | stage i |
| 305 | TCGA.BP.4807.01A | high | 211 | 42 | Right | G3 | M0 | NX | T1a | TUMOR FREE | male | stage i |
| 306 | TCGA.BP.4959.01A | low | 2660 | 49 | Right | G3 | M0 | NX | T1b | TUMOR FREE | male | stage i |
| 307 | TCGA.BP.4960.01A | low | 2172 | 46 | Left | G3 | M0 | N0 | T2 | TUMOR FREE | male | stage ii |
| 308 | TCGA.BP.4961.01A | high | 1935 | 47 | Right | G2 | M0 | NX | T1a | TUMOR FREE | male | stage i |
| 309 | TCGA.BP.4962.01A | low | 1785 | 58 | Left | G2 | M0 | NX | T2 | TUMOR FREE | male | stage ii |
| 310 | TCGA.BP.4963.01A | high | 1834 | 63 | Left | G3 | M0 | NX | T1b | TUMOR FREE | male | stage i |
| 311 | TCGA.BP.4964.01A | high | 1862 | 54 | Right | G2 | M0 | N0 | T1a | TUMOR FREE | female | stage i |
| 312 | TCGA.BP.4965.01A | high | 1871 | 46 | Left | G2 | M0 | NX | T1a | TUMOR FREE | male | stage i |
| 313 | TCGA.BP.4967.01A | high | 205 | 76 | Right | G2 | M0 | N0 | T3a | TUMOR FREE | male | stage iii |
| 314 | TCGA.BP.4968.01A | high | 1746 | 40 | Right | G3 | M0 | N0 | T1b | TUMOR FREE | male | stage i |
| 315 | TCGA.BP.4969.01A | low | 1794 | 63 | Right | G2 | M0 | NX | T1a | TUMOR FREE | female | stage i |
| 316 | TCGA.BP.4970.01A | high | 433 | 44 | Right | G3 | M0 | N1 | T1a | TUMOR FREE | male | stage iii |
| 317 | TCGA.BP.4971.01A | low | 1487 | 40 | Right | G3 | M0 | N0 | T3a | TUMOR FREE | male | stage iii |
| 318 | TCGA.BP.4972.01A | low | 1502 | 43 | Left | G3 | M0 | NX | T3a | TUMOR FREE | female | stage iii |
| 319 | TCGA.BP.4973.01A | high | 1384 | 47 | Right | G3 | M0 | NX | T3a | TUMOR FREE | male | stage iii |
| 320 | TCGA.BP.4974.01A | high | 211 | 58 | Right | G4 | M1 | N0 | T3a | WITH TUMOR | male | stage iv |
| 321 | TCGA.BP.4975.01A | high | 1433 | 40 | Right | G3 | M0 | NX | T1b | TUMOR FREE | male | stage i |
| 322 | TCGA.BP.4976.01A | high | 1632 | 77 | Right | G3 | M0 | NX | T1a | TUMOR FREE | male | stage i |
| 323 | TCGA.BP.4977.01A | low | 454 | 57 | Left | G3 | M0 | NX | T1b | TUMOR FREE | male | stage i |
| 324 | TCGA.BP.4981.01A | low | 1097 | 75 | Right | G3 | M0 | NX | T3a | TUMOR FREE | female | stage iii |
| 325 | TCGA.BP.4982.01A | high | 1014 | 42 | Left | G3 | M0 | NX | T1b | TUMOR FREE | male | stage i |
| 326 | TCGA.BP.4983.01A | low | 1413 | 67 | Right | G4 | M0 | NX | T3a | TUMOR FREE | female | stage iii |
| 327 | TCGA.BP.4985.01A | low | 952 | 72 | Left | G4 | M0 | N0 | T3a | WITH TUMOR | male | stage iii |
| 328 | TCGA.BP.4986.01A | low | 785 | 75 | Right | G3 | M0 | N0 | T1a | TUMOR FREE | male | stage i |
| 329 | TCGA.BP.4987.01A | high | 1124 | 41 | Left | G2 | M0 | NX | T1b | TUMOR FREE | female | stage i |
| 330 | TCGA.BP.4989.01A | low | 118 | 58 | Right | G3 | M0 | N0 | T3a | TUMOR FREE | male | stage iii |
| 331 | TCGA.BP.4991.01A | high | 1413 | 54 | Right | G2 | M0 | NX | T1a | TUMOR FREE | male | stage i |
| 332 | TCGA.BP.4992.01A | low | 501 | 66 | Left | G4 | M0 | NX | T1b | TUMOR FREE | male | stage i |
| 333 | TCGA.BP.4993.01A | low | 177 | 58 | Left | G3 | M0 | NX | T1a | TUMOR FREE | male | stage i |
| 334 | TCGA.BP.4994.01A | high | 1308 | 54 | Left | G3 | M0 | NX | T1a | TUMOR FREE | male | stage i |
| 335 | TCGA.BP.4995.01A | high | 1371 | 68 | Right | G3 | M0 | N0 | T1b | TUMOR FREE | male | stage i |
| 336 | TCGA.BP.4998.01A | high | 932 | 49 | Right | G3 | M0 | NX | T1a | TUMOR FREE | male | stage i |
| 337 | TCGA.BP.4999.01A | high | 1266 | 56 | Left | G2 | M0 | NX | T1a | TUMOR FREE | male | stage i |
| 338 | TCGA.BP.5000.01A | low | 563 | 40 | Left | G3 | M0 | NX | T1b | TUMOR FREE | male | stage i |
| 339 | TCGA.BP.5001.01A | low | 1177 | 43 | Right | G2 | M0 | NX | T1b | TUMOR FREE | female | stage i |
| 340 | TCGA.BP.5004.01A | high | 1126 | 53 | Right | G3 | M0 | NX | T1a | TUMOR FREE | male | stage i |
| 341 | TCGA.BP.5006.01A | low | 840 | 61 | Left | G2 | M0 | N0 | T1a | TUMOR FREE | male | stage i |
| 342 | TCGA.BP.5007.01A | high | 1140 | 45 | Left | G2 | M0 | N0 | T2 | TUMOR FREE | male | stage ii |
| 343 | TCGA.BP.5008.01A | low | 1071 | 46 | Right | G2 | M0 | NX | T1a | TUMOR FREE | male | stage i |
| 344 | TCGA.BP.5009.01A | low | 1092 | 52 | Left | G3 | M0 | NX | T1b | WITH TUMOR | male | stage i |
| 345 | TCGA.BP.5010.01A | low | 878 | 63 | Right | G4 | M0 | N0 | T3a | WITH TUMOR | male | stage iii |
| 346 | TCGA.BP.5168.01A | high | 1463 | 75 | Right | G2 | M0 | NX | T1a | TUMOR FREE | male | stage i |
| 347 | TCGA.BP.5169.01A | low | 193 | 70 | Left | G4 | M0 | N0 | T1b | TUMOR FREE | male | stage i |
| 348 | TCGA.BP.5170.01A | low | 2412 | 55 | Right | G2 | M0 | NX | T1a | TUMOR FREE | male | stage i |
| 349 | TCGA.BP.5173.01A | high | 62 | 75 | Right | G2 | M0 | NX | T1a | TUMOR FREE | male | stage i |
| 350 | TCGA.BP.5174.01A | low | 2257 | 45 | Left | G2 | M0 | NX | T1a | TUMOR FREE | female | stage i |
| 351 | TCGA.BP.5175.01A | low | 932 | 60 | Right | G3 | M0 | NX | T1a | TUMOR FREE | male | stage i |
| 352 | TCGA.BP.5176.01A | low | 1590 | 78 | Left | G2 | M0 | NX | T1a | TUMOR FREE | female | stage i |
| 353 | TCGA.BP.5177.01A | low | 293 | 46 | Right | G3 | M0 | NX | T1a | TUMOR FREE | female | stage i |
| 354 | TCGA.BP.5178.01A | low | 1912 | 71 | Right | G4 | M1 | NX | T3a | WITH TUMOR | male | stage iv |
| 355 | TCGA.BP.5180.01A | low | 2263 | 53 | Right | G2 | M0 | NX | T1a | TUMOR FREE | male | stage i |
| 356 | TCGA.BP.5181.01A | low | 1495 | 58 | Left | G2 | M0 | NX | T1b | TUMOR FREE | female | stage i |
| 357 | TCGA.BP.5182.01A | low | 1165 | 56 | Left | G3 | M0 | N0 | T1a | TUMOR FREE | male | stage i |
| 358 | TCGA.BP.5183.01A | high | 1291 | 57 | Left | G3 | M0 | NX | T3a | TUMOR FREE | male | stage iii |
| 359 | TCGA.BP.5184.01A | high | 1133 | 54 | Left | G3 | M0 | NX | T1a | TUMOR FREE | male | stage i |
| 360 | TCGA.BP.5185.01A | low | 1132 | 56 | Right | G3 | M0 | NX | T1a | WITH TUMOR | male | stage i |
| 361 | TCGA.BP.5186.01A | high | 693 | 50 | Right | G2 | M0 | N0 | T1a | TUMOR FREE | female | stage i |
| 362 | TCGA.BP.5187.01A | low | 406 | 54 | Right | G2 | M0 | NX | T1a | TUMOR FREE | male | stage i |
| 363 | TCGA.BP.5189.01A | high | 822 | 60 | Right | G4 | M0 | NX | T1b | WITH TUMOR | male | stage i |
| 364 | TCGA.BP.5190.01A | low | 1011 | 61 | Right | G3 | M0 | NX | T1a | TUMOR FREE | male | stage i |
| 365 | TCGA.BP.5191.01A | low | 967 | 79 | Left | G2 | M0 | N0 | T3a | TUMOR FREE | male | stage iii |
| 366 | TCGA.BP.5192.01A | high | 714 | 59 | Right | G2 | M0 | NX | T1a | TUMOR FREE | male | stage i |
| 367 | TCGA.BP.5194.01A | high | 408 | 39 | Right | G2 | M0 | NX | T1a | TUMOR FREE | male | stage i |
| 368 | TCGA.BP.5195.01A | low | 749 | 75 | Left | G2 | M0 | NX | T1a | TUMOR FREE | male | stage i |
| 369 | TCGA.BP.5196.01A | high | 1018 | 53 | Left | G2 | M0 | NX | T1a | TUMOR FREE | male | stage i |
| 370 | TCGA.BP.5198.01A | high | 603 | 72 | Right | G3 | M0 | N0 | T3b | TUMOR FREE | male | stage iii |
| 371 | TCGA.BP.5199.01A | low | 1355 | 58 | Left | G4 | M0 | N0 | T2 | TUMOR FREE | male | stage ii |
| 372 | TCGA.BP.5200.01A | low | 1063 | 44 | Right | G4 | M0 | NX | T2 | TUMOR FREE | male | stage ii |
| 373 | TCGA.BP.5201.01A | low | 951 | 63 | Left | G4 | M1 | N0 | T3b | WITH TUMOR | male | stage iv |
| 374 | TCGA.BP.5202.01A | high | 29 | 75 | Right | G2 | M0 | NX | T3a | TUMOR FREE | male | stage iii |
| 375 | TCGA.CJ.4634.01A | high | 3498 | 60 | Right | G2 | M0 | NX | T1b | TUMOR FREE | female | stage i |
| 376 | TCGA.CJ.4635.01A | high | 1416 | 48 | Left | G3 | M0 | NX | T1b | TUMOR FREE | male | stage i |
| 377 | TCGA.CJ.4636.01A | low | 1924 | 51 | Right | G3 | M0 | N0 | T3a | TUMOR FREE | male | stage iii |
| 378 | TCGA.CJ.4637.01A | low | 2227 | 52 | Left | G4 | M1 | NX | T2b | WITH TUMOR | female | stage iv |
| 379 | TCGA.CJ.4638.01A | low | 431 | 46 | Left | G4 | M1 | N1 | T3a | WITH TUMOR | female | stage iv |
| 380 | TCGA.CJ.4639.01A | high | 3229 | 49 | Left | G2 | M0 | N0 | T2 | TUMOR FREE | female | stage ii |
| 381 | TCGA.CJ.4640.01A | high | 3480 | 49 | Left | G4 | M0 | N0 | T3a | TUMOR FREE | male | stage iii |
| 382 | TCGA.CJ.4641.01A | high | 1661 | 55 | Left | G4 | M1 | NX | T3a | WITH TUMOR | female | stage iv |
| 383 | TCGA.CJ.4643.01A | high | 1793 | 67 | Right | G3 | M0 | N0 | T2b | TUMOR FREE | female | stage ii |
| 384 | TCGA.CJ.4644.01A | high | 336 | 48 | Left | G3 | M1 | N0 | T3a | WITH TUMOR | female | stage iv |
| 385 | TCGA.CJ.4868.01A | low | 646 | 42 | Left | G3 | M1 | N0 | T3a | WITH TUMOR | male | stage iv |
| 386 | TCGA.CJ.4869.01A | low | 2554 | 49 | Right | G2 | M0 | N1 | T2 | WITH TUMOR | male | stage iii |
| 387 | TCGA.CJ.4870.01A | low | 1498 | 58 | Left | G2 | M0 | NX | T3a | TUMOR FREE | female | stage iii |
| 388 | TCGA.CJ.4871.01A | low | 2423 | 63 | Left | G4 | M1 | NX | T3a | TUMOR FREE | male | stage iv |
| 389 | TCGA.CJ.4872.01A | low | 1435 | 51 | Right | G4 | M0 | N0 | T1b | TUMOR FREE | male | stage i |
| 390 | TCGA.CJ.4873.01A | low | 1776 | 85 | Right | G3 | M0 | N0 | T3a | TUMOR FREE | female | stage iii |
| 391 | TCGA.CJ.4874.01A | high | 2283 | 73 | Left | G3 | M0 | N0 | T1b | TUMOR FREE | female | stage i |
| 392 | TCGA.CJ.4875.01A | low | 3554 | 67 | Right | G3 | M1 | NX | T3a | WITH TUMOR | male | stage iv |
| 393 | TCGA.CJ.4876.01A | high | 1955 | 57 | Left | G3 | M0 | N0 | T2b | TUMOR FREE | male | stage ii |
| 394 | TCGA.CJ.4878.01A | high | 2186 | 71 | Left | G2 | M0 | NX | T3a | TUMOR FREE | female | stage iii |
| 395 | TCGA.CJ.4881.01A | low | 2014 | 41 | Right | G3 | M0 | NX | T3a | WITH TUMOR | male | stage iii |
| 396 | TCGA.CJ.4882.01A | low | 1883 | 57 | Right | G3 | M0 | NX | T3a | TUMOR FREE | male | stage iii |
| 397 | TCGA.CJ.4884.01A | low | 1759 | 72 | Right | G3 | M0 | NX | T3a | TUMOR FREE | female | stage iii |
| 398 | TCGA.CJ.4885.01A | low | 3451 | 64 | Right | G3 | M1 | NX | T3a | WITH TUMOR | male | stage iv |
| 399 | TCGA.CJ.4886.01A | high | 1952 | 42 | Left | G3 | M0 | NX | T1a | TUMOR FREE | female | stage i |
| 400 | TCGA.CJ.4887.01A | low | 932 | 48 | Right | G3 | M1 | NX | T3a | WITH TUMOR | male | stage iv |
| 401 | TCGA.CJ.4888.01A | low | 1567 | 59 | Left | G4 | M1 | NX | T3a | WITH TUMOR | male | stage iv |
| 402 | TCGA.CJ.4889.01A | low | 1946 | 63 | Left | G4 | M0 | NX | T1a | TUMOR FREE | female | stage i |
| 403 | TCGA.CJ.4890.01A | low | 3519 | 72 | Left | G4 | M1 | N0 | T3a | WITH TUMOR | male | stage iv |
| 404 | TCGA.CJ.4891.01A | low | 819 | 57 | Left | G4 | M0 | N0 | T3c | TUMOR FREE | female | stage iii |
| 405 | TCGA.CJ.4892.01A | high | 1521 | 65 | Right | G2 | M0 | N0 | T1b | TUMOR FREE | female | stage i |
| 406 | TCGA.CJ.4893.01A | high | 750 | 76 | Right | G3 | M0 | NX | T1b | TUMOR FREE | female | stage i |
| 407 | TCGA.CJ.4894.01A | high | 841 | 58 | Left | G3 | M0 | N0 | T3a | WITH TUMOR | male | stage iii |
| 408 | TCGA.CJ.4895.01A | low | 1200 | 62 | Left | G4 | M1 | NX | T3a | WITH TUMOR | male | stage iv |
| 409 | TCGA.CJ.4897.01A | high | 3341 | 79 | Right | G3 | M0 | NX | T3a | WITH TUMOR | female | stage iii |
| 410 | TCGA.CJ.4899.01A | high | 1528 | 42 | Left | G2 | M0 | NX | T1b | TUMOR FREE | male | stage i |
| 411 | TCGA.CJ.4900.01A | low | 1714 | 69 | Left | G4 | M1 | N1 | T4 | WITH TUMOR | female | stage iv |
| 412 | TCGA.CJ.4901.01A | low | 1450 | 47 | Right | G3 | M0 | NX | T3b | TUMOR FREE | male | stage iii |
| 413 | TCGA.CJ.4902.01A | low | 1520 | 61 | Right | G3 | M0 | NX | T3a | TUMOR FREE | male | stage iii |
| 414 | TCGA.CJ.4903.01A | low | 1560 | 50 | Left | G3 | M0 | NX | T1b | TUMOR FREE | male | stage i |
| 415 | TCGA.CJ.4904.01A | high | 3302 | 60 | Left | G3 | M1 | N0 | T3a | WITH TUMOR | female | stage iv |
| 416 | TCGA.CJ.4905.01A | high | 1496 | 62 | Right | G2 | M0 | NX | T1a | TUMOR FREE | female | stage i |
| 417 | TCGA.CJ.4907.01A | high | 1499 | 58 | Right | G3 | M0 | NX | T3b | TUMOR FREE | male | stage iii |
| 418 | TCGA.CJ.4908.01A | low | 1531 | 38 | Right | G2 | M0 | NX | T1a | TUMOR FREE | male | stage i |
| 419 | TCGA.CJ.4912.01A | low | 1657 | 61 | Left | G3 | M0 | NX | T2 | TUMOR FREE | male | stage ii |
| 420 | TCGA.CJ.4916.01A | low | 1373 | 69 | Right | G3 | M0 | NX | T3a | TUMOR FREE | female | stage iii |
| 421 | TCGA.CJ.4918.01A | low | 93 | 64 | Left | G4 | M1 | N0 | T3a | WITH TUMOR | male | stage iv |
| 422 | TCGA.CJ.4920.01A | low | 139 | 64 | Left | G2 | M0 | NX | T1b | TUMOR FREE | female | stage i |
| 423 | TCGA.CJ.5671.01A | low | 3987 | 51 | Left | G3 | M0 | NX | T1a | TUMOR FREE | male | stage i |
| 424 | TCGA.CJ.5672.01A | low | 1972 | 84 | Left | G3 | M0 | NX | T1a | TUMOR FREE | male | stage i |
| 425 | TCGA.CJ.5675.01A | low | 3936 | 70 | Right | G3 | M0 | NX | T2a | WITH TUMOR | male | stage ii |
| 426 | TCGA.CJ.5676.01A | low | 4067 | 47 | Right | G3 | M0 | NX | T3b | WITH TUMOR | male | stage iii |
| 427 | TCGA.CJ.5677.01A | low | 782 | 54 | Right | G4 | M1 | NX | T3a | WITH TUMOR | female | stage iv |
| 428 | TCGA.CJ.5678.01A | low | 574 | 62 | Left | G3 | M1 | N0 | T2b | WITH TUMOR | male | stage iv |
| 429 | TCGA.CJ.5679.01A | low | 679 | 73 | Left | G4 | M0 | NX | T3b | WITH TUMOR | male | stage iii |
| 430 | TCGA.CJ.5680.01A | high | 768 | 65 | Right | G4 | M1 | NX | T3a | WITH TUMOR | female | stage iv |
| 431 | TCGA.CJ.5681.01A | high | 552 | 44 | Left | G3 | M1 | NX | T3a | WITH TUMOR | female | stage iv |
| 432 | TCGA.CJ.5682.01A | high | 3736 | 60 | Left | G4 | M1 | NX | T3a | WITH TUMOR | male | stage iv |
| 433 | TCGA.CJ.5683.01A | high | 1889 | 78 | Right | G3 | M0 | NX | T1b | TUMOR FREE | male | stage i |
| 434 | TCGA.CJ.5684.01A | high | 2231 | 61 | Right | G2 | M0 | NX | T3a | TUMOR FREE | male | stage iii |
| 435 | TCGA.CJ.5686.01A | high | 2038 | 59 | Left | G3 | M0 | NX | T1b | TUMOR FREE | female | stage i |
| 436 | TCGA.CJ.6027.01A | low | 3615 | 77 | Right | G4 | M0 | NX | T1a | TUMOR FREE | male | stage i |
| 437 | TCGA.CJ.6028.01A | low | 1625 | 58 | Left | G4 | M1 | NX | T3a | WITH TUMOR | male | stage iv |
| 438 | TCGA.CJ.6030.01A | low | 2299 | 65 | Left | G3 | M0 | N0 | T1a | TUMOR FREE | male | stage i |
| 439 | TCGA.CJ.6031.01A | low | 1906 | 54 | Right | G3 | M0 | NX | T1b | TUMOR FREE | male | stage i |
| 440 | TCGA.CJ.6032.01A | low | 3639 | 63 | Right | G3 | M0 | NX | T2 | TUMOR FREE | female | stage ii |
| 441 | TCGA.CJ.6033.01A | low | 224 | 54 | Right | G4 | M1 | N0 | T3a | WITH TUMOR | female | stage iv |
| 442 | TCGA.CW.5580.01A | low | 1964 | 73 | Right | G3 | M1 | NX | T3a |  | female | stage iv |
| 443 | TCGA.CW.5581.01A | high | 2799 | 44 | Right | G3 | M0 | NX | T1b | TUMOR FREE | male | stage i |
| 444 | TCGA.CW.5583.01A | high | 2489 | 51 | Right | G2 | M0 | NX | T1a | TUMOR FREE | female | stage i |
| 445 | TCGA.CW.5584.01A | high | 164 | 74 | Right | G3 | M0 | N1 | T3b |  | male | stage iii |
| 446 | TCGA.CW.5585.01A | high | 2609 | 51 | Right | G2 | M1 | N0 | T3b | WITH TUMOR | male | stage iv |
| 447 | TCGA.CW.5587.01A | high | 2226 | 62 | Left | G2 | M0 | N0 | T3b | TUMOR FREE | female | stage iii |
| 448 | TCGA.CW.5588.01A | low | 2017 | 78 | Right | G2 | M0 | NX | T1a |  | female | stage i |
| 449 | TCGA.CW.5589.01A | high | 2378 | 52 | Left | G2 | M0 | NX | T1a | TUMOR FREE | male | stage i |
| 450 | TCGA.CW.5590.01A | high | 1075 | 51 | Right | G3 | M1 | NX | T3a |  | male | stage iv |
| 451 | TCGA.CW.5591.01A | high | 2271 | 56 | Right | G2 | M1 | N0 | T3a | WITH TUMOR | male | stage iv |
| 452 | TCGA.CW.6087.01A | low | 41 | 61 | Right | G4 | M1 | N1 | T3a | TUMOR FREE | male | stage iv |
| 453 | TCGA.CW.6088.01A | high | 3222 | 60 | Right | G2 | M0 | N0 | T1b |  | male | stage i |
| 454 | TCGA.CW.6090.01A | high | 2552 | 68 | Right | G3 | M0 | NX | T1b |  | male | stage i |
| 455 | TCGA.CW.6093.01A | high | 3146 | 73 | Right | G1 | M0 | NX | T1a |  | male | stage i |
| 456 | TCGA.CW.6097.01A | high | 571 | 32 | Left | G4 | M0 | NX | T3a | WITH TUMOR | male | stage iii |
| 457 | TCGA.CZ.4853.01A | high | 774 | 82 | Right | G2 | M0 | NX | T1a | TUMOR FREE | male | stage i |
| 458 | TCGA.CZ.4854.01A | low | 1404 | 68 | Right | G2 | M0 | N0 | T1b | TUMOR FREE | male | stage i |
| 459 | TCGA.CZ.4856.01A | low | 18 | 62 | Left | G2 | M0 | N0 | T1b |  | female | stage i |
| 460 | TCGA.CZ.4857.01A | low | 1432 | 56 | Right | G3 | M1 | N0 | T3a | WITH TUMOR | male | stage iv |
| 461 | TCGA.CZ.4858.01A | low | 2105 | 39 | Left | G4 | M0 | NX | T2 | WITH TUMOR | male | stage ii |
| 462 | TCGA.CZ.4859.01A | high | 1787 | 59 | Right | G2 | M0 | N0 | T1 | TUMOR FREE | female | stage i |
| 463 | TCGA.CZ.4860.01A | low | 206 | 60 | Right | G4 | M1 | NX | T4 | WITH TUMOR | male | stage iv |
| 464 | TCGA.CZ.4861.01A | low | 446 | 63 | Left | G2 | M0 | NX | T2 | WITH TUMOR | male | stage ii |
| 465 | TCGA.CZ.4862.01A | high | 3271 | 46 | Left | G2 | M0 | NX | T1b | TUMOR FREE | male | stage i |
| 466 | TCGA.CZ.4863.01A | low | 1928 | 51 | Left | G3 | M0 | N0 | T3b | TUMOR FREE | female | stage iii |
| 467 | TCGA.CZ.4864.01A | high | 1315 | 86 | Left | G3 | M0 | N0 | T2 | TUMOR FREE | male | stage ii |
| 468 | TCGA.CZ.4865.01A | high | 166 | 70 | Left | G2 | M0 | NX | T1a | TUMOR FREE | female | stage i |
| 469 | TCGA.CZ.4866.01A | high | 3267 | 79 | Right | G3 | M0 | NX | T1 | TUMOR FREE | female | stage i |
| 470 | TCGA.CZ.5451.01A | high | 1929 | 74 | Left | G3 | M0 | N0 | T2 | TUMOR FREE | male | stage ii |
| 471 | TCGA.CZ.5452.01A | high | 1789 | 69 | Right | G2 | M0 | N0 | T2 | TUMOR FREE | male | stage ii |
| 472 | TCGA.CZ.5453.01A | low | 2419 | 67 | Left | G2 | M0 | NX | T2 |  | male | stage ii |
| 473 | TCGA.CZ.5454.01A | low | 722 | 63 | Right | G2 | M1 | N0 | T2 | WITH TUMOR | male | stage iv |
| 474 | TCGA.CZ.5455.01A | high | 561 | 63 | Right | G4 | M1 | NX | T3b | WITH TUMOR | male | stage iv |
| 475 | TCGA.CZ.5456.01A | low | 2422 | 57 | Left | G3 | M0 | N0 | T2 | WITH TUMOR | male | stage ii |
| 476 | TCGA.CZ.5457.01A | high | 2754 | 62 | Right | G4 | M0 | NX | T3a | WITH TUMOR | male | stage iii |
| 477 | TCGA.CZ.5458.01A | high | 2789 | 43 | Right | G3 | M0 | NX | T3a | TUMOR FREE | male | stage iii |
| 478 | TCGA.CZ.5459.01A | low | 1683 | 63 | Right | G3 | M0 | NX | T3b | TUMOR FREE | male | stage iii |
| 479 | TCGA.CZ.5460.01A | high | 2873 | 55 | Right | G2 | M1 | NX | T3b | WITH TUMOR | male | stage iv |
| 480 | TCGA.CZ.5461.01A | high | 330 | 52 | Right | G4 | M1 | NX | T1b | WITH TUMOR | male | stage iv |
| 481 | TCGA.CZ.5462.01A | high | 311 | 83 | Right | G3 | M1 | NX | T1b | WITH TUMOR | male | stage iv |
| 482 | TCGA.CZ.5463.01A | high | 662 | 76 | Left | G2 | M0 | NX | T2 |  | male | stage ii |
| 483 | TCGA.CZ.5464.01A | low | 2128 | 69 | Left | G2 | M1 | NX | T3b | WITH TUMOR | male | stage iv |
| 484 | TCGA.CZ.5465.01A | high | 2564 | 76 | Left | G2 | M0 | NX | T3b | TUMOR FREE | female | stage iii |
| 485 | TCGA.CZ.5466.01A | low | 685 | 67 | Right | G2 | M0 | NX | T3a | TUMOR FREE | male | stage iii |
| 486 | TCGA.CZ.5467.01A | high | 73 | 86 | Right | G4 | M0 | N0 | T3a | WITH TUMOR | female | stage iii |
| 487 | TCGA.CZ.5468.01A | low | 59 | 84 | Left | G4 | M1 | NX | T3b | WITH TUMOR | male | stage iv |
| 488 | TCGA.CZ.5469.01A | low | 946 | 41 | Left | G2 | M0 | N0 | T2 | WITH TUMOR | male | stage ii |
| 489 | TCGA.CZ.5470.01A | high | 386 | 72 | Left | G3 | M0 | N0 | T2 |  | female | stage ii |
| 490 | TCGA.CZ.5982.01A | high | 2439 | 59 | Left | G2 | M0 | NX | T1a | TUMOR FREE | female | stage i |
| 491 | TCGA.CZ.5984.01A | high | 2067 | 51 | Left | G3 | M0 | N0 | T1b | TUMOR FREE | male | stage i |
| 492 | TCGA.CZ.5985.01A | low | 1997 | 58 | Right | G2 | M0 | N0 | T2 | TUMOR FREE | male | stage ii |
| 493 | TCGA.CZ.5986.01A | high | 373 | 61 | Left | G3 | M0 | N0 | T1 | TUMOR FREE | male | stage i |
| 494 | TCGA.CZ.5987.01A | low | 445 | 60 | Right | G2 | M1 | NX | T3b | WITH TUMOR | male | stage iv |
| 495 | TCGA.CZ.5988.01A | low | 693 | 38 | Right | G2 | M0 | N0 | T1b | TUMOR FREE | male | stage i |
| 496 | TCGA.CZ.5989.01A | high | 1905 | 60 | Right | G2 | M0 | N0 | T2 | TUMOR FREE | male | stage ii |
| 497 | TCGA.DV.5565.01A | low | 1329 | 59 | Right | G2 | M0 | NX | T1a | WITH TUMOR | male | stage i |
| 498 | TCGA.DV.5566.01A | high | 1398 | 67 | Right | G2 | M0 | NX | T1a | WITH TUMOR | female | stage i |
| 499 | TCGA.DV.5567.01A | high | 2004 | 40 | Right | G2 | M0 | NX | T1a | WITH TUMOR | female | stage i |
| 500 | TCGA.DV.5568.01A | high | 370 | 26 | Right | G2 | M0 | NX | T1a | WITH TUMOR | male | stage i |
| 501 | TCGA.DV.5569.01A | high | 355 | 29 | Right | G2 | M0 | NX | T1a | TUMOR FREE | female | stage i |
| 502 | TCGA.DV.5573.01A | high | 1130 | 41 | Right | G2 | M0 | NX | T1a | WITH TUMOR | male | stage i |
| 503 | TCGA.DV.5574.01A | low | 2016 | 37 | Right | G2 | M0 | NX | T1a | WITH TUMOR | male | stage i |
| 504 | TCGA.DV.5575.01A | high | 1729 | 52 | Left | G2 | M0 | NX | T1a | WITH TUMOR | female | stage i |
| 505 | TCGA.DV.5576.01A | high | 727 | 55 | Left | G2 | M0 | NX | T1a | WITH TUMOR | female | stage i |
| 506 | TCGA.DV.A4VX.01A | low | 1626 | 59 | Right | G4 | MX | N0 | T3b | WITH TUMOR | male | stage iv |
| 507 | TCGA.DV.A4VZ.01A | high | 365 | 53 | Right | G2 | MX | NX | T1a |  | male | stage i |
| 508 | TCGA.DV.A4W0.01A | low | 2470 | 55 | Left | G3 | MX | NX | T1b | WITH TUMOR | male | stage i |
| 509 | TCGA.EU.5904.01A | high | 551 | 47 | Right | G1 | M0 | NX | T1 | TUMOR FREE | female | stage i |
| 510 | TCGA.EU.5905.01A | high | 119 | 67 | Right | G3 | M0 | NX | T1 | TUMOR FREE | female | stage i |
| 511 | TCGA.EU.5906.01A | high | 206 | 55 | Left | G2 | M0 | NX | T1b | TUMOR FREE | male | stage i |
| 512 | TCGA.EU.5907.01A | high | 127 | 81 | Right | G3 | M0 | NX | T3a | TUMOR FREE | male | stage iii |
| 513 | TCGA.G6.A5PC.01A | low | 242 | 54 | Left | G4 | M1 | N0 | T1b |  | female | stage iv |
| 514 | TCGA.G6.A8L6.01A | low | 313 | 55 | Left | G3 | MX | NX | T2a | WITH TUMOR | male | stage iv |
| 515 | TCGA.G6.A8L7.01A | low | 2133 | 81 | Right | G3 | MX | N0 | T1b | TUMOR FREE | female | stage i |
| 516 | TCGA.G6.A8L8.01A | high | 1091 | 62 | Right | G3 | MX | NX | T1b |  | female | stage i |
| 517 | TCGA.GK.A6C7.01A | high | 61 | 76 | Right |  | MX | NX | T1a | TUMOR FREE | female | stage i |
| 518 | TCGA.MM.A563.01A | high | 591 | 41 | Left | G2 | MX | NX | T3 | TUMOR FREE | male | not reported |
| 519 | TCGA.MM.A564.01A | low | 607 | 68 | Right | G2 | MX | NX | T2a | TUMOR FREE | male | stage ii |
| 520 | TCGA.MM.A84U.01A | low | 700 | 58 |  | G2 | MX | NX | T1a | TUMOR FREE | female | stage i |
| 521 | TCGA.MW.A4EC.01A | low | 498 | 72 | Right | G2 | MX | NX | T1a | TUMOR FREE | female | stage i |
| 522 | TCGA.T7.A92I.01A | high | 356 | 47 | Right | G1 | MX | NX | T1a | TUMOR FREE | female | stage i |
| 523 | TCGA.B8.4146.01B | high | 511 | 41 | Right | G2 | M0 | NX | T1b | TUMOR FREE | female | stage i |
| 524 | TCGA.B8.4153.01B | low | 762 | 74 | Left | G3 | M0 | NX | T3a | WITH TUMOR | male | stage iii |
| 525 | TCGA.B8.5552.01B | high | 1046 | 41 | Right | G2 | M0 | NX | T1b | TUMOR FREE | female | stage i |
| 526 | TCGA.CJ.4642.01B | high | 3205 | 47 | Right | G2 | M0 | NX | T2 | TUMOR FREE | male | stage ii |
