# Supplementary material for: Integrated bioinformatics analysis of the NEDD4 family reveals a prognostic value of NEDD4L in clear-cell renal cell cancer
Source: PeerJ. 2021 Aug 17;9:e11880. doi: 10.7717/peerj.11880 (PMC8378337; doi:10.7717/peerj.11880)
Supplement: Supplemental Information 7 [file peerj-09-11880-s007.docx]

Supplementary table 2 NEDD4L-related genes

| Symbol | beta | HR (95% CI for HR) | wald.test | p.value |
| --- | --- | --- | --- | --- |
| SOWAHB | -0.5 | 0.6 (0.54-0.68) | 69 | 8.50E-17 |
| WDR72 | -0.38 | 0.69 (0.63-0.75) | 63 | 1.90E-15 |
| EPB41L4A_DT | -0.77 | 0.46 (0.38-0.56) | 58 | 3.10E-14 |
| C4orf19 | -0.53 | 0.59 (0.51-0.68) | 57 | 4.40E-14 |
| RBM47 | -0.64 | 0.53 (0.44-0.62) | 56 | 8.00E-14 |
| AKAP6 | -0.86 | 0.42 (0.33-0.54) | 51 | 7.90E-13 |
| ALDH6A1 | -0.46 | 0.63 (0.55-0.72) | 49 | 3.00E-12 |
| NR3C2 | -0.53 | 0.59 (0.51-0.68) | 48 | 3.30E-12 |
| SLC25A4 | -0.69 | 0.5 (0.42-0.61) | 48 | 3.70E-12 |
| CDKL2 | -0.62 | 0.54 (0.45-0.64) | 47 | 5.60E-12 |
| PCCA | -0.54 | 0.58 (0.5-0.68) | 47 | 5.70E-12 |
| ACAT1 | -0.45 | 0.64 (0.56-0.73) | 43 | 5.80E-11 |
| PLCL2 | -0.53 | 0.59 (0.5-0.69) | 43 | 6.40E-11 |
| IRF6 | -0.35 | 0.71 (0.64-0.78) | 42 | 7.80E-11 |
| ETFDH | -0.69 | 0.5 (0.41-0.62) | 42 | 7.90E-11 |
| CRY2 | -0.53 | 0.59 (0.5-0.69) | 42 | 8.00E-11 |
| HSPA4L | -0.55 | 0.58 (0.49-0.68) | 42 | 1.00E-10 |
| RNF152 | -0.43 | 0.65 (0.57-0.74) | 41 | 1.30E-10 |
| AUH | -0.57 | 0.56 (0.47-0.67) | 41 | 1.40E-10 |
| HSD17B8 | -0.61 | 0.54 (0.45-0.65) | 41 | 1.50E-10 |
| BAG1 | -0.75 | 0.47 (0.37-0.59) | 41 | 1.60E-10 |
| KBTBD3 | -0.82 | 0.44 (0.34-0.57) | 41 | 1.90E-10 |
| ASTN2 | -0.65 | 0.52 (0.43-0.64) | 40 | 2.10E-10 |
| PPARGC1A | -0.41 | 0.67 (0.59-0.76) | 40 | 2.90E-10 |
| TOLLIP | -0.75 | 0.47 (0.37-0.6) | 40 | 3.20E-10 |
| TJP2 | -0.51 | 0.6 (0.51-0.71) | 39 | 3.50E-10 |
| CDS1 | -0.46 | 0.63 (0.55-0.73) | 39 | 3.90E-10 |
| MPP5 | -0.57 | 0.57 (0.47-0.68) | 38 | 6.30E-10 |
| TRIM2 | -0.46 | 0.63 (0.54-0.73) | 38 | 7.60E-10 |
| NUDT7 | -0.76 | 0.47 (0.36-0.59) | 38 | 9.10E-10 |
| ENPP4 | -0.43 | 0.65 (0.56-0.75) | 37 | 1.20E-09 |
| CCDC121 | -0.7 | 0.5 (0.39-0.62) | 36 | 2.00E-09 |
| MEGF9 | -0.47 | 0.63 (0.54-0.73) | 35 | 3.10E-09 |
| CAT | -0.41 | 0.67 (0.58-0.76) | 35 | 3.60E-09 |
| CTDSPL | -0.57 | 0.56 (0.47-0.68) | 35 | 4.10E-09 |
| NCR3LG1 | -0.42 | 0.65 (0.57-0.75) | 34 | 4.50E-09 |
| ZC2HC1C | -0.78 | 0.46 (0.35-0.6) | 34 | 7.00E-09 |
| COQ7 | -0.86 | 0.42 (0.32-0.57) | 33 | 7.70E-09 |
| MPP7 | -0.61 | 0.54 (0.44-0.67) | 33 | 7.90E-09 |
| GOT1 | -0.48 | 0.62 (0.52-0.73) | 33 | 8.30E-09 |
| MYO6 | -0.45 | 0.64 (0.55-0.74) | 33 | 8.40E-09 |
| NBEA | -0.52 | 0.6 (0.5-0.71) | 33 | 9.70E-09 |
| SCAPER | -0.58 | 0.56 (0.46-0.68) | 33 | 1.10E-08 |
| CPT1A | -0.48 | 0.62 (0.52-0.73) | 33 | 1.10E-08 |
| SYNJ2BP | -0.59 | 0.56 (0.46-0.68) | 33 | 1.10E-08 |
| LIN7A | -0.28 | 0.76 (0.69-0.83) | 32 | 1.50E-08 |
| ATP6V1D | -0.68 | 0.51 (0.4-0.64) | 32 | 1.80E-08 |
| SLC30A9 | -0.51 | 0.6 (0.5-0.72) | 32 | 1.80E-08 |
| EDA | -0.55 | 0.58 (0.48-0.7) | 32 | 1.90E-08 |
| FBXO34 | -0.53 | 0.59 (0.49-0.71) | 31 | 2.20E-08 |
| SOS2 | -0.56 | 0.57 (0.47-0.69) | 31 | 2.70E-08 |
| NKIRAS1 | -0.7 | 0.5 (0.39-0.64) | 31 | 2.80E-08 |
| PLEKHA7 | -0.4 | 0.67 (0.58-0.77) | 30 | 3.90E-08 |
| ZNF844 | -0.46 | 0.63 (0.53-0.74) | 30 | 4.50E-08 |
| KIF13B | -0.52 | 0.59 (0.49-0.72) | 30 | 4.70E-08 |
| DLAT | -0.54 | 0.58 (0.48-0.71) | 30 | 5.30E-08 |
| PPM1A | -0.55 | 0.58 (0.47-0.7) | 30 | 5.50E-08 |
| MYO5B | -0.46 | 0.63 (0.53-0.75) | 29 | 5.90E-08 |
| GPAT3 | -0.35 | 0.7 (0.62-0.8) | 29 | 8.50E-08 |
| NDFIP1 | -0.65 | 0.52 (0.41-0.66) | 29 | 9.00E-08 |
| ERMP1 | -0.48 | 0.62 (0.52-0.74) | 28 | 1.30E-07 |
| LGR4 | -0.38 | 0.68 (0.59-0.79) | 28 | 1.30E-07 |
| L2HGDH | -0.49 | 0.61 (0.51-0.73) | 28 | 1.40E-07 |
| ACSS3 | -0.34 | 0.71 (0.62-0.81) | 28 | 1.50E-07 |
| SECISBP2L | -0.5 | 0.6 (0.5-0.73) | 27 | 1.90E-07 |
| TTC39B | -0.57 | 0.57 (0.46-0.7) | 27 | 2.10E-07 |
| CA2 | -0.33 | 0.72 (0.64-0.82) | 27 | 2.20E-07 |
| PHKB | -0.63 | 0.53 (0.42-0.68) | 27 | 2.20E-07 |
| PRMT9 | -0.63 | 0.53 (0.42-0.68) | 27 | 2.40E-07 |
| GNA11 | -0.53 | 0.59 (0.48-0.72) | 27 | 2.40E-07 |
| AP5M1 | -0.68 | 0.51 (0.39-0.66) | 27 | 2.60E-07 |
| KBTBD4 | -0.48 | 0.62 (0.52-0.74) | 27 | 2.60E-07 |
| MTURN | -0.43 | 0.65 (0.55-0.77) | 26 | 2.70E-07 |
| ATP10D | -0.44 | 0.65 (0.55-0.77) | 26 | 4.10E-07 |
| TOX3 | -0.33 | 0.72 (0.64-0.82) | 26 | 4.20E-07 |
| BPGM | -0.6 | 0.55 (0.43-0.69) | 25 | 5.10E-07 |
| OGDHL | -0.24 | 0.79 (0.72-0.86) | 25 | 5.40E-07 |
| OSBP | -0.52 | 0.6 (0.49-0.73) | 25 | 5.60E-07 |
| GPD1L | -0.51 | 0.6 (0.49-0.73) | 25 | 6.00E-07 |
| SC5D | -0.48 | 0.62 (0.51-0.75) | 25 | 6.10E-07 |
| TBC1D1 | -0.6 | 0.55 (0.43-0.69) | 25 | 6.80E-07 |
| UGT8 | -0.35 | 0.7 (0.61-0.81) | 24 | 1.00E-06 |
| TACC2 | -0.54 | 0.58 (0.47-0.72) | 24 | 1.00E-06 |
| ZNF468 | -0.52 | 0.59 (0.48-0.73) | 24 | 1.00E-06 |
| HSD11B2 | -0.25 | 0.78 (0.71-0.86) | 24 | 1.20E-06 |
| ARL6 | -0.53 | 0.59 (0.47-0.73) | 23 | 1.40E-06 |
| MPPED2 | -0.68 | 0.51 (0.38-0.67) | 23 | 1.50E-06 |
| TBC1D14 | -0.35 | 0.71 (0.61-0.81) | 23 | 1.50E-06 |
| PTPN3 | -0.38 | 0.69 (0.59-0.8) | 23 | 1.60E-06 |
| SDHD | -0.45 | 0.64 (0.53-0.77) | 23 | 1.70E-06 |
| ZNF684 | -0.64 | 0.53 (0.41-0.69) | 23 | 2.10E-06 |
| ATP6V1A | -0.41 | 0.67 (0.56-0.79) | 22 | 2.20E-06 |
| JADE1 | -0.64 | 0.53 (0.4-0.69) | 22 | 2.30E-06 |
| PPFIBP2 | -0.44 | 0.64 (0.54-0.77) | 22 | 2.50E-06 |
| SCAMP1 | -0.45 | 0.63 (0.53-0.77) | 22 | 2.50E-06 |
| PEX7 | -0.53 | 0.59 (0.47-0.73) | 22 | 2.80E-06 |
| PIGO | -0.45 | 0.64 (0.53-0.77) | 22 | 3.10E-06 |
| GPHN | -0.44 | 0.64 (0.53-0.77) | 22 | 3.40E-06 |
| SBF2 | -0.43 | 0.65 (0.55-0.78) | 22 | 3.50E-06 |
| DDB1 | -0.58 | 0.56 (0.44-0.72) | 21 | 3.80E-06 |
| FREM1 | -0.55 | 0.58 (0.45-0.73) | 21 | 4.20E-06 |
| SPTLC2 | -0.47 | 0.63 (0.51-0.76) | 21 | 4.30E-06 |
| IDH3A | -0.43 | 0.65 (0.54-0.78) | 21 | 5.90E-06 |
| SCOC | -0.52 | 0.6 (0.48-0.75) | 20 | 6.40E-06 |
| LARGE2 | -0.25 | 0.78 (0.7-0.87) | 20 | 6.90E-06 |
| CD9 | -0.33 | 0.72 (0.62-0.83) | 20 | 7.40E-06 |
| SNX13 | -0.48 | 0.62 (0.5-0.77) | 20 | 8.40E-06 |
| ATP5F1B | -0.47 | 0.63 (0.51-0.77) | 20 | 8.70E-06 |
| ISCA2 | -0.6 | 0.55 (0.42-0.72) | 19 | 1.20E-05 |
| LIMA1 | -0.41 | 0.66 (0.55-0.8) | 19 | 1.40E-05 |
| IVD | -0.51 | 0.6 (0.47-0.76) | 19 | 1.70E-05 |
| ST7 | -0.58 | 0.56 (0.43-0.73) | 18 | 1.80E-05 |
| MKKS | -0.63 | 0.53 (0.4-0.71) | 18 | 1.90E-05 |
| EXPH5 | -0.44 | 0.64 (0.52-0.79) | 18 | 1.90E-05 |
| CIPC | -0.4 | 0.67 (0.56-0.81) | 18 | 2.00E-05 |
| GLRX5 | -0.59 | 0.56 (0.42-0.73) | 18 | 2.10E-05 |
| DGLUCY | -0.53 | 0.59 (0.46-0.75) | 18 | 2.40E-05 |
| DLG3 | -0.55 | 0.58 (0.45-0.75) | 18 | 2.80E-05 |
| TMEM72 | -0.18 | 0.84 (0.77-0.91) | 17 | 2.90E-05 |
| KIAA1328 | -0.66 | 0.52 (0.38-0.71) | 17 | 3.00E-05 |
| TMEM8B | -0.41 | 0.66 (0.54-0.8) | 17 | 3.00E-05 |
| SH2D4A | -0.4 | 0.67 (0.55-0.81) | 17 | 3.10E-05 |
| PTPN21 | -0.43 | 0.65 (0.53-0.8) | 17 | 3.40E-05 |
| ADAL | -0.64 | 0.53 (0.39-0.71) | 17 | 3.40E-05 |
| MYRIP | -0.36 | 0.7 (0.59-0.83) | 17 | 4.00E-05 |
| DHRS7 | -0.51 | 0.6 (0.47-0.76) | 17 | 4.00E-05 |
| DPH6 | -0.78 | 0.46 (0.32-0.66) | 17 | 4.00E-05 |
| RAP1GAP | -0.26 | 0.77 (0.69-0.88) | 17 | 4.50E-05 |
| HDAC11 | -0.53 | 0.59 (0.45-0.76) | 16 | 5.10E-05 |
| LARS2 | -0.44 | 0.64 (0.52-0.8) | 16 | 5.70E-05 |
| KLHL26 | -0.57 | 0.57 (0.43-0.75) | 16 | 6.20E-05 |
| SIRT5 | -0.54 | 0.58 (0.44-0.76) | 16 | 6.90E-05 |
| DMAC2L | -0.51 | 0.6 (0.46-0.77) | 16 | 7.60E-05 |
| MUC20 | -0.22 | 0.8 (0.72-0.9) | 16 | 8.10E-05 |
| NUDT9 | -0.54 | 0.58 (0.44-0.76) | 16 | 8.10E-05 |
| NIPAL1 | -0.58 | 0.56 (0.42-0.75) | 15 | 8.80E-05 |
| STRBP | -0.51 | 0.6 (0.47-0.78) | 15 | 0.00011 |
| KIF21A | -0.31 | 0.73 (0.63-0.86) | 15 | 0.00011 |
| TSG101 | -0.54 | 0.59 (0.45-0.77) | 15 | 0.00012 |
| PDCD6IP | -0.46 | 0.63 (0.5-0.8) | 15 | 0.00012 |
| CCNB1IP1 | -0.39 | 0.68 (0.55-0.83) | 15 | 0.00013 |
| GPRC5B | -0.28 | 0.76 (0.66-0.88) | 14 | 0.00015 |
| ARFGEF2 | -0.33 | 0.72 (0.6-0.85) | 14 | 0.00018 |
| ATPAF1 | -0.54 | 0.58 (0.44-0.77) | 14 | 2.00E-04 |
| ACAD8 | -0.48 | 0.62 (0.48-0.8) | 14 | 0.00022 |
| RDH11 | -0.45 | 0.64 (0.5-0.81) | 13 | 0.00025 |
| RAB11FIP4 | -0.51 | 0.6 (0.46-0.79) | 13 | 0.00027 |
| BCKDHB | -0.35 | 0.7 (0.58-0.85) | 13 | 0.00029 |
| PDP2 | -0.53 | 0.59 (0.44-0.78) | 13 | 0.00029 |
| OXCT1 | -0.28 | 0.75 (0.65-0.88) | 13 | 3.00E-04 |
| COL4A3 | -0.27 | 0.77 (0.66-0.89) | 13 | 0.00038 |
| SECISBP2 | -0.37 | 0.69 (0.56-0.85) | 12 | 0.00041 |
| NSUN7 | -0.32 | 0.72 (0.6-0.87) | 12 | 0.00047 |
| SLC16A11 | -0.37 | 0.69 (0.56-0.86) | 11 | 0.00079 |
| TMEM38A | -0.32 | 0.72 (0.6-0.87) | 11 | 0.00081 |
| KRBA2 | -0.65 | 0.52 (0.36-0.77) | 11 | 0.00083 |
| RAB40B | -0.37 | 0.69 (0.55-0.86) | 11 | 0.0011 |
| CWH43 | -0.34 | 0.71 (0.58-0.87) | 10 | 0.0013 |
| PCNX4 | -0.39 | 0.68 (0.53-0.86) | 9.9 | 0.0017 |
| ZNF664 | -0.34 | 0.71 (0.58-0.88) | 9.8 | 0.0018 |
| CHCHD4 | -0.53 | 0.59 (0.42-0.83) | 9.3 | 0.0023 |
| SLC25A12 | -0.46 | 0.63 (0.47-0.85) | 9 | 0.0027 |
| ZNF91 | -0.38 | 0.68 (0.53-0.88) | 8.5 | 0.0035 |
| ZDHHC3 | -0.38 | 0.68 (0.53-0.88) | 8.4 | 0.0036 |
| ATP2B2 | -0.19 | 0.83 (0.73-0.94) | 8.5 | 0.0036 |
| PRDM4 | -0.33 | 0.72 (0.58-0.9) | 8.4 | 0.0037 |
| TM7SF2 | -0.25 | 0.78 (0.65-0.92) | 8.2 | 0.0041 |
| FOXI2 | -0.36 | 0.7 (0.54-0.9) | 7.9 | 0.0051 |
| GGT6 | -0.24 | 0.79 (0.67-0.93) | 7.6 | 0.0058 |
| LINC00472 | -0.28 | 0.75 (0.61-0.92) | 7.5 | 0.006 |
| ACACB | -0.28 | 0.75 (0.61-0.92) | 7.4 | 0.0066 |
| ILF3_DT | -0.27 | 0.76 (0.62-0.93) | 6.9 | 0.0085 |
| FAM189A2 | -0.26 | 0.77 (0.63-0.95) | 6.1 | 0.014 |
| TMEM101 | -0.3 | 0.74 (0.58-0.94) | 6 | 0.014 |
| C3orf18 | -0.27 | 0.76 (0.61-0.95) | 5.9 | 0.016 |
| RALGPS1 | -0.32 | 0.73 (0.55-0.96) | 5.2 | 0.022 |
| TMEM213 | -0.14 | 0.87 (0.77-0.98) | 5.1 | 0.024 |
| ATP6V0A4 | -0.12 | 0.88 (0.79-0.99) | 4.7 | 0.03 |
| NUPR2 | -0.19 | 0.83 (0.69-0.99) | 4.4 | 0.035 |
| NRG2 | -0.31 | 0.73 (0.55-0.98) | 4.4 | 0.037 |
| INSYN1 | -0.22 | 0.81 (0.66-0.99) | 4.2 | 0.04 |
| ZDHHC23 | -0.2 | 0.81 (0.67-0.99) | 4.1 | 0.043 |
| ARNT2 | -0.14 | 0.87 (0.76-1) | 4.1 | 0.043 |
