# Supplementary material for: Integrated bioinformatics analysis of the NEDD4 family reveals a prognostic value of NEDD4L in clear-cell renal cell cancer
Source: PeerJ. 2021 Aug 17;9:e11880. doi: 10.7717/peerj.11880 (PMC8378337; doi:10.7717/peerj.11880)
Supplement: Supplemental Information 8 [file peerj-09-11880-s008.docx]

Supplementary table 3 GO enrich of NEDD4L

| Description | pvalue | p.adjust | qvalue | Count |
| --- | --- | --- | --- | --- |
| pyrophosphate hydrolysis-driven proton transmembrane transporter activity | 1.58E-05 | 0.003887 | 0.00346 | 5 |
| ATPase-coupled cation transmembrane transporter activity | 2.00E-05 | 0.003887 | 0.00346 | 6 |
| ATPase-coupled ion transmembrane transporter activity | 3.03E-05 | 0.003887 | 0.00346 | 6 |
| ATPase activity, coupled to transmembrane movement of ions, rotational mechanism | 4.83E-05 | 0.003887 | 0.00346 | 4 |
| proton-transporting ATPase activity, rotational mechanism | 4.83E-05 | 0.003887 | 0.00346 | 4 |
| ATPase-coupled transmembrane transporter activity | 8.39E-05 | 0.005619 | 0.005003 | 7 |
| primary active transmembrane transporter activity | 0.000129 | 0.007381 | 0.006571 | 7 |
| NAD binding | 0.000264 | 0.013284 | 0.011826 | 5 |
| palmitoyltransferase activity | 0.000703 | 0.031415 | 0.027969 | 4 |
| quinone binding | 0.000807 | 0.032442 | 0.028882 | 3 |
| mitochondrial matrix | 1.22E-09 | 3.42E-07 | 3.14E-07 | 22 |
| proton-transporting two-sector ATPase complex | 1.06E-05 | 0.001481 | 0.001359 | 6 |
| proton-transporting V-type ATPase complex | 0.000125 | 0.011625 | 0.010663 | 4 |
| apical junction complex | 0.000487 | 0.034101 | 0.031281 | 7 |
| integral component of mitochondrial membrane | 0.001131 | 0.048115 | 0.044136 | 5 |
| intrinsic component of mitochondrial membrane | 0.001195 | 0.048115 | 0.044136 | 5 |
| bicellular tight junction | 0.001203 | 0.048115 | 0.044136 | 6 |
| small molecule catabolic process | 2.10E-08 | 4.84E-05 | 4.40E-05 | 20 |
| branched-chain amino acid catabolic process | 5.37E-08 | 6.18E-05 | 5.62E-05 | 6 |
| branched-chain amino acid metabolic process | 1.24E-07 | 9.55E-05 | 8.68E-05 | 6 |
| fatty acid catabolic process | 1.85E-06 | 0.001063 | 0.000967 | 9 |
| organic acid catabolic process | 4.43E-06 | 0.001703 | 0.001548 | 13 |
| carboxylic acid catabolic process | 4.43E-06 | 0.001703 | 0.001548 | 13 |
| fatty acid beta-oxidation | 8.05E-06 | 0.002501 | 0.002274 | 7 |
| monocarboxylic acid catabolic process | 8.92E-06 | 0.002501 | 0.002274 | 9 |
| fatty acid oxidation | 9.77E-06 | 0.002501 | 0.002274 | 8 |
| lipid oxidation | 1.37E-05 | 0.003161 | 0.002874 | 8 |
